# Cytosine base editor-DNA binding domain fusions for editing window modulation in the RNP format

**DOI:** 10.1186/s12896-025-01020-1

**Published:** 2025-08-29

**Authors:** Erin Brettmann, Fuqiang Chen, Stephen Beishir, Graeme Garvey

**Affiliations:** 1 Merck KGaA, Darmstadt, Germany; 2 St. Louis, MO USA; 3 Present Address: Corteva, Johnston, IA USA

**Keywords:** CRISPR, Cas9, Base editing, Cytosine base editor, Precision editing, RNP

## Abstract

Base editing technologies allow for the precise and efficient installation of defined nucleotide substitutions into a target genome without the introduction of double strand breaks or DNA templates. Here we describe two recombinant, protein format cytosine base editors (CBEs) that efficiently catalyze the installation of cytosine-to-thymine edits, termed “Flexible” and “Precision.” Flexible exhibits a wide editing window, while Precision uses a fused single-stranded DNA binding protein to narrow the editing window, lowering the risk of editing multiple cytosine residues at the target site. We show that co-transfection with uracil glycosylase inhibitor protein increases the proportion of substitutions that are C-to-T and the ratio of C-to-T editing to indel formation, thus reducing undesired editing outcomes. We use in vitro editing assays to characterize our editors and show a preference for cytosine residues preceded by thymine (TpC dinucleotides) and unmethylated cytosine residues.

**Supplementary Information:**

The online version contains supplementary material available at 10.1186/s12896-025-01020-1.

## Introduction

CRISPR-Cas9 systems have revolutionized the field of genome engineering, allowing researchers to introduce a wide range of modifications in the genome. In the technology’s most simple form, base pairing between a guide RNA and a genomic target leads to Cas9 protein binding and cleavage of double stranded DNA (dsDNA). This interaction requires the presence of a protospacer adjacent motif (PAM), a short sequence that defines a potential target sequence. For the most commonly used Cas9 protein, SpCas9, this PAM is a 5’-NGG-3’ trinucleotide immediately downstream of the guide-binding site. This technology readily introduces insertions and deletions (indels) into a targeted sequence via the non-homologous end joining (NHEJ) pathway, resulting in gene inactivation. However, precision editing, or the modification of a target to a specific, predetermined sequence, requires additional components. This can be achieved via homology directed repair (HDR), in which a donor DNA serves as a template to repair the double strand break (DSB) introduced by Cas9 [1, 2]. However, there are several challenges inherent to HDR. In particular, the mammalian NHEJ pathway is more efficient than HDR [3], and as a result, HDR rates tend to be low and unintended indel outcomes are frequent. Further, the template donor DNA itself is toxic in some cell types and may integrate nonspecifically into the genome [4, 5].

An alternative precision editing method is base editing, wherein a catalytic effector domain fused to a Cas nuclease domain chemically modifies a targeted nucleobase, resulting in a substitution. In the case of cytosine base editing, a deaminase domain fused to Cas9 allows for the installation of C-to-T substitutions at the targeted sequence. Notably, this technology does not require a DNA donor and it uses Cas proteins with fully or partially inactivated endonuclease domains, resulting in a lower level of DSBs and their associated indel repair outcomes. Base editors were developed initially by the Liu lab [6, 7] and have become a widely used tool in a diverse range of organisms, from bacteria to plants to humans, even entering clinical trials as a therapeutic to correct pathogenic mutations. Notably, a base editor was recently used in vivo to correct a fatal genetic condition in a human infant, with marked clinical improvement in the patient [8], reported to be the first direct correction of a genetic disease in a human.

There remain several challenges to base editing that may limit its use in a research or therapeutic context. One challenge is the so-called “bystander editing,” where nucleotides at the target site other than the intended nucleotide are edited. To address this, researchers have engineered base editor proteins with rigid linkers [9], constrained the activity of the deaminase domain to specific sequence contexts [10, 11], or reduced the frequency at which multiple residues are edited at a single target site [12]. Each of these approaches carries its own limitation; constraining editing to specific positions within the target and constraining editing to specific sequence contexts each limit which sequences are editable in different ways.

Another challenge with cytosine base editing, specifically, is a result of repair by the base excision repair (BER) pathway. In cytosine base editing, cytosine is deaminated to uracil, which serves to template the installation of a thymidine residue following DNA repair and/or replication. Uracil does not naturally occur in DNA; rather, it signifies DNA damage, either from misincorporation or spontaneous deamination of cytosine, both of which are mutagenic. Uracil DNA glycosylase detects deoxyuridine and excises the uracil base, and the resultant abasic site is repaired using the opposite (undamaged) strand as a template [13]. In the context of base editing, this serves to “correct” the edit, reverting the target to the unedited sequence. Beyond reducing editing efficiency, BER intermediates can themselves be mutagenic. Replication of an abasic site by low-fidelity translesion polymerases can introduce undesired C-to-A and C-to-G substitutions, and the intrinsic fragility of abasic sites can lead to DNA backbone breakage [14]. In the context of cytosine base editors (CBEs) with Cas9 nickase activity, this can lead to DSBs and their indel repair products. To minimize these undesirable BER outcomes, CBEs typically include 1–2 uracil glycosylase inhibitor (UGI) domains fused to the C terminus of protein, which effectively increase the rate of C-to-T substitution while reducing the rates of C-to-A and C-to-G substitutions and indels [6, 15].

A final challenge to base editing is the predominance of plasmid delivery of base editing components. The ease of plasmid delivery of CBE components and the high rates of editing have made the plasmid delivery format ubiquitous. However, plasmid delivery has several drawbacks. It increases the risk of off-target effects, including editing of similar sequences (“mismatch editing”) and genome-wide nonspecific deamination of cytosines, due to extended overexpression of the base editor [16, 17]. Plasmid DNA is toxic in some cell types, as seen with donor DNA for HDR editing. Plasmid DNA may, at low frequency, integrate into the host genome. These drawbacks serve as a barrier to the use of CBEs, particularly in the therapeutic space. Expression and purification of CBE protein from *E. coli* is challenging because a high level of deaminase expression is typically toxic to bacterial cells. Additionally, successful purification of CBE proteins requires specialized equipment and expertise, including sonicators and FPLC (fast protein liquid chromatography) instruments that may not be available to every laboratory [16, 18, 19].

There is a need for a commercially available protein format CBE that can be expressed and purified at commercial scale and that, when delivered as a ribonucleoprotein (RNP), results in high rates of C-to-T editing with low rates of off-target editing and undesired repair products. Jang et al. reported expression and purification from immortalized mammalian cells as a method to circumvent the difficulty in purifying these proteins [18]; however, large-scale purification from mammalian cells is not cost-effective. Here we describe the development of CBEs that meet the aforementioned criteria. We show that fusion of a phage-derived single-stranded DNA binding protein (SSB) to the N-terminus of the CBE results in a narrowed editing window. We show that covalent attachment of UGI is insufficient to suppress BER and lowers editing rates when the CBE is delivered in the RNP format, but that this can be remedied by co-delivery of recombinant UGI protein. Finally, we show that transfection of CBE RNPs alongside a commercially available CRISPR enhancer yields high rates of C-to-T editing with lower off-target effects than seen with plasmid delivery. Together, these tools will enable more labs to harness the advantages of RNP CBE delivery.

## Results & discussion

### Screening for CBE variants with narrowed editing windows

**Fig. 1:**
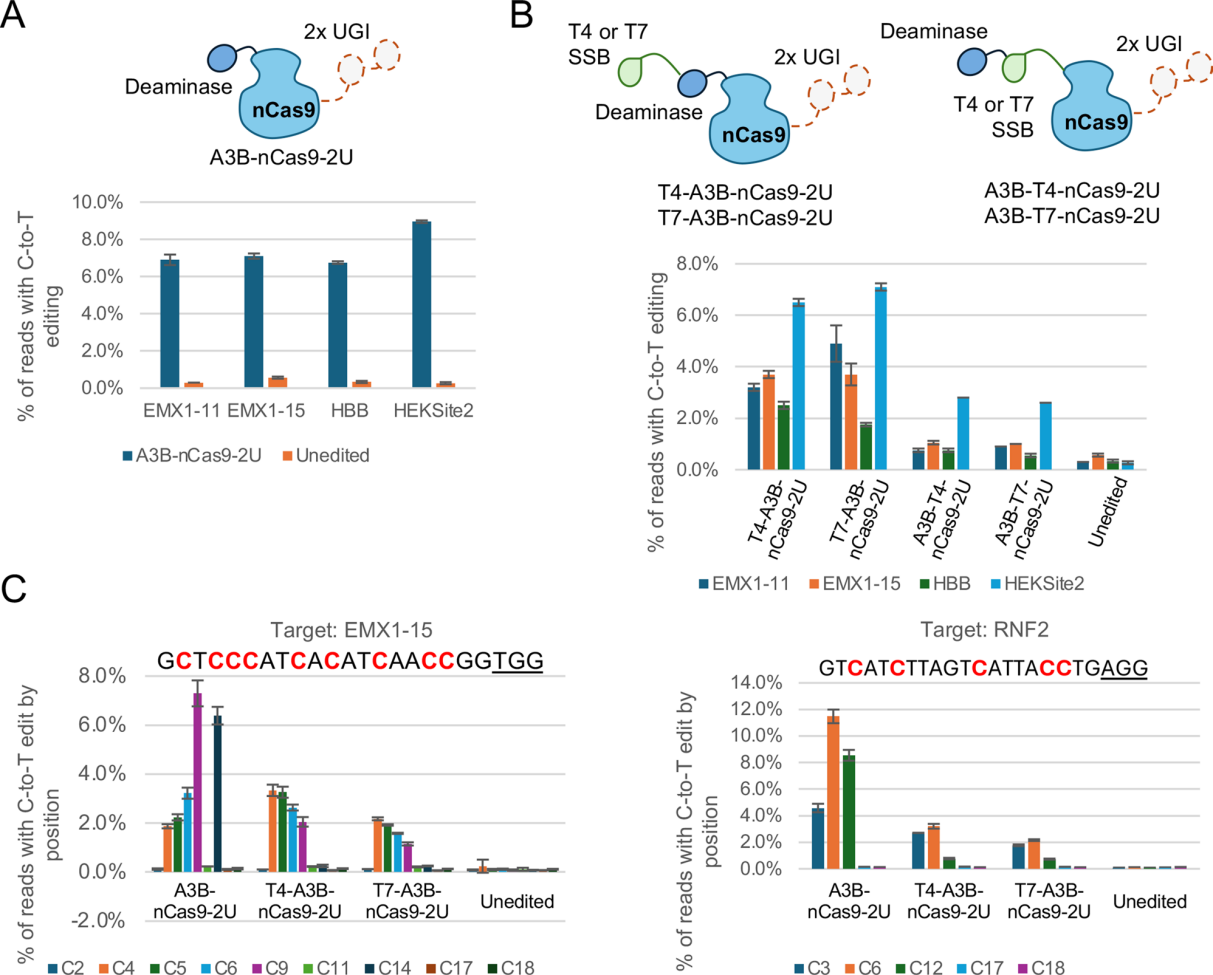
Screening of CBE variant proteins identifies active variants with different editing windows. Proteins were purified using gravity column chromatography. **A**) C-to-T editing rates in HEK293 cells for the A3B-nCas9-2U CBE protein at four genomic targets. Data for A3B-nCas9-2U are the average of two technical replicates and data for the unedited control are the average of three biological replicates. Error bars represent the standard deviation. **B**) C-to-T editing rates in HEK293 cells for the CBE proteins with viral SSB domains at four genomic targets. Data for CBE samples are the average of two technical replicates and data for the unedited control are the average of three biological replicates. Error bars represent the standard deviation. T4 and T7 variants were transfected in separate experiments. **C**) C-to-T editing rates by position at the genomic targets EMX1-15 and RNF2 in HEK293 cells for CBE variants A3B-nCas9-2U, T4–A3B-nCas9-2U, and T7–A3B-nCas9-2U. The target site is indicated above the graph, with the PAM sequence underlined and cytosine residues in red. Cytosine positions are numbered from the 5’ end of the target sequence. Data for CBE samples are the average of two technical replicates and data for the unedited control are the average of four biological replicates. Error bars represent the standard deviation. All CBE proteins were transfected in the same experiment. See also Figs. S1 and S2

We sought to engineer a CBE variant that could be readily purified from *E. coli* and had a narrowed editing window. To achieve this, we first designed a CBE variant following the architecture of BE4 [15] that used the C-terminal domain of human APOBEC3B (A3B) as the deaminase domain and expressed and purified it from *E. coli.* In essence, this variant consisted of an SpCas9 nickase with the deaminase domain tethered to the N-terminus and two UGI domains tethered to the C-terminus using flexible peptide linkers (Table S1). Using a simplified purification scheme of immobilized metal affinity chromatography (IMAC), HRV-3C tag cleavage, dialysis, and secondary IMAC tag clean up, we achieved a yield of approximately 0.5 mg protein per gram of cell paste. In contrast, we had observed in preliminary experiments that CBEs containing rat ABOBEC1 or *Petromyzon marinus* CDA1 expressed poorly, and mainly as truncations. To test the level of C-to-T editing conferred by this CBE, we complexed it with chemically synthesized single guide RNAs (sgRNAs) targeting genomic sites and nucleofected the RNPs into HEK293 cells. We found that the CBE resulted in measurable base editing, as evaluated by next-generation sequencing (NGS) (Fig. 1A).

An A3B-nCas9 CBE for plasmid delivery has been reported previously and was found to have a relatively wide editing window [12]. This is useful in situations with restricted PAM availability as it allows for more flexible placement of the guide relative to the target residue, but less useful for targets with nearby cytosine residues that should remain unedited. To change the base editing window to a more defined, narrowed position within the target, we hypothesized that fusion of a protein domain with single stranded DNA binding activity would occlude a portion of the target sequence and render it unavailable for base editing, thereby narrowing the editing window. To investigate this, we tested the effect of adding a viral single stranded DNA binding protein (SSB) to the basic base editor. We selected T4 bacteriophage Gp32 and T7 bacteriophage Gp2.5 for their existing thorough characterization at the protein level, small size (~34 and ~25 kDa, respectively), and monomeric binding to DNA, and investigated the effects of adding monomeric domains to the basic base editor architecture at different positions (Table S1): 1) fusion of the viral SSB to the N-terminus of the base editor; and 2) fusion of the viral SSB between the deaminase and nCas9. Purification of these variants yielded 0.5–1 mg protein per gram of cell paste, sufficient to enable production at scale.

As shown in Fig. 1B, fusion proteins containing the SSB domain display editing activity well above background when the SSB was located at the N-terminus of the fusion protein. However, when the SSB was located between the deaminase and the nCas9, most targets are minimally edited. These results differ from previously published works that report fusions of CBE with SSB domains. In one report, Komor et al. found that the fusion of SSB to the C-terminus of a CBE resulted in a loss of editing activity [15]; we found that placement of the SSB at the N-terminus allows for efficient editing. Our results also differ from those of Zhang et al. [20], who report that the fusion of the Rad51 DNA-binding domain between the deaminase and nCas9 domains results in increased editing efficiency with a wider editing window. In contrast, we find that this arrangement results in reduced editing efficiency. The differences between our results and theirs may be due to the particular SSB domains used (viral vs human Rad51) or to differences in delivery method (RNP vs plasmid).

We then evaluated the positions within the target sites that were edited by the unmodified, basic CBE proteins and the SSB-containing CBE proteins (Fig. 1C and Fig. S1). We found that the N-terminal SSB fusion proteins maintained editing at positions in the PAM-distal region of the target but had reduced or absent editing at more PAM-proximal positions, indicating a narrowed editing window, regardless of whether the SSB was derived from T4 or T7 bacteriophage. We termed these N-terminal-SSB-containing CBEs “Precision” editors for their ability to discriminate between nearby cytosine residues and the basic CBE “Flexible” editor for its ability to edit at more positions within the target. The overall editing rate of the T4 SSB variant was significantly higher than that of the T7 SSB variant for three of the four genomic sites tested (Fig. S2); therefore, we proceeded with the T4 SSB variant for further development.

### Further editing improvement through standalone uracil glycosylase inhibitor and transfection enhancer PEXBUFF

The presence of uracil in DNA is often the consequence of DNA damage. The base excision repair (BER) pathway uses the enzyme uracil DNA glycosylase (UDG) to identify and excise uracil nucleobases from DNA, resulting in an abasic site, which is then repaired. In the context of cytosine base editing, the action of BER results in the reversion of successful edits to the unedited state. In addition, it can lead to the installation of C-to-R substitutions and indels at the site of the excised uracil nucleobase. To combat these undesired outcomes, cytosine base editors typically have one or more fused uracil glycosylase inhibitor (UGI) domains. UGI irreversibly binds to the cellular UDG, preventing it from binding and excising deoxyuridine residues [21]. As a result, cytosine base editors containing these UGI domains have an increased rate of C-to-T substitutions and a reduced rate of C-to-R substitutions and indels [6, 15]. However, Jang et al. found that UGI activity in CBEs delivered as proteins was insufficient to effect meaningful UDG inhibition [18]; instead, they delivered UGI expressed from a plasmid.

**Fig. 2:**
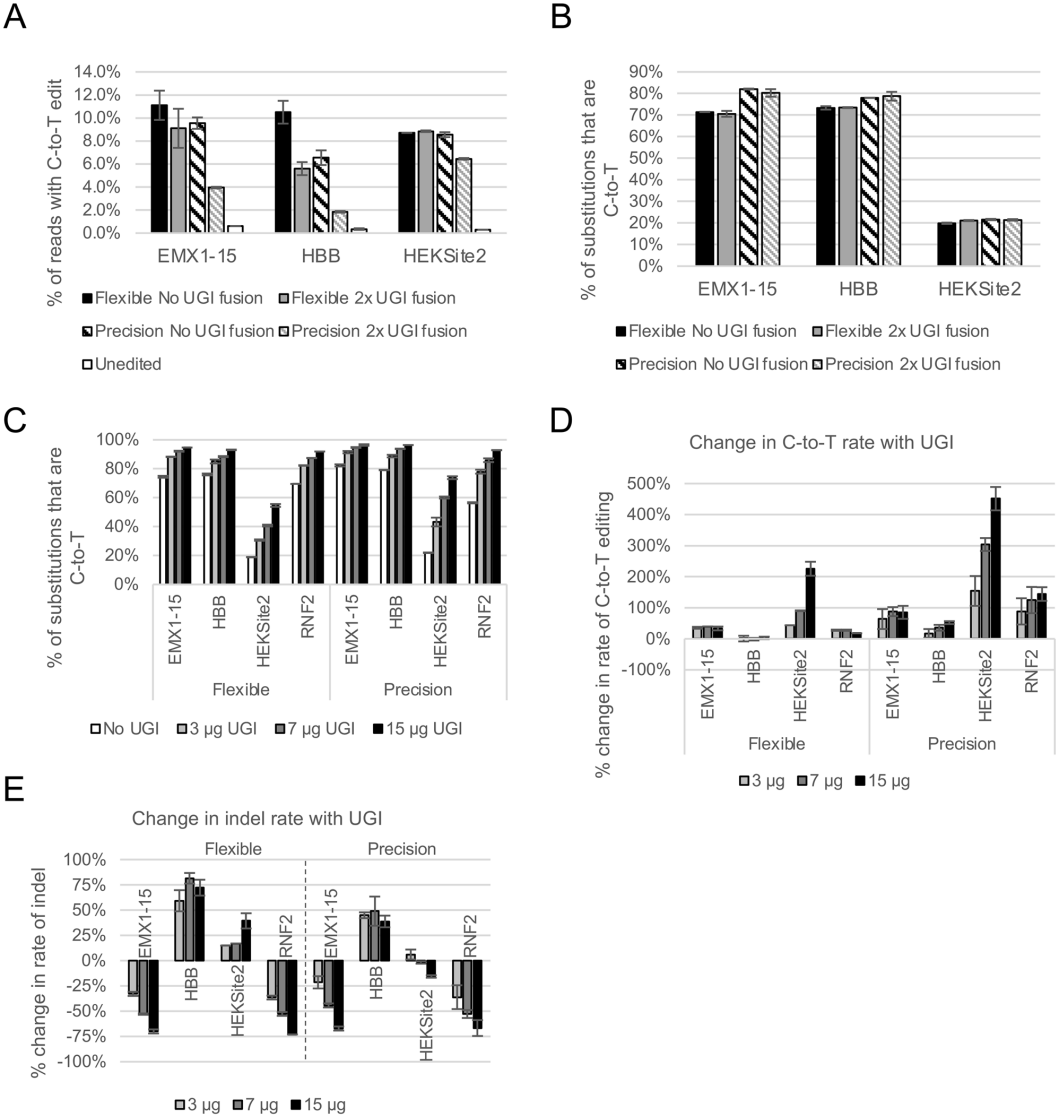
Fused UGI domains are insufficient to improve the rate and proportion of C-to-T edits. Proteins were purified using gravity column chromatography. **A**) C-to-T editing rates in HEK293 cells at three genomic targets for Flexible (solid bars) and Precision (hashed bars) with (gray) and without (black) 2x UGI as part of the fusion protein. Data for CBE samples are the average of two technical replicates and data for the unedited control are the average of two biological replicates. Error bars represent the standard deviation. Flexible and Precision variants were transfected in separate experiments. **B**) Percent of substitutions that are C-to-T in HEK293 cells at three genomic targets for Flexible (solid bars) and Precision (hashed bars) with (gray) and without (black) 2x UGI as part of the fusion protein. Analyses for (A) and (B) use the same read data set. See also Fig. S3. **C**) Percent of substitutions that are C-to-T across all target site cytosine residues for four genomic targets in transfections that omit UGI protein (white bars) or with increasing amounts of co-delivered UGI protein (gray to black bars). **D**) Percent change in the rate of C-to-T substitution with co-delivery of UGI at four genomic sites. Values are in comparison to transfections that omit UGI protein. E) Percent change in the rate of indel formation with co-delivery of UGI protein at four genomic sites. Values are in comparison to transfections that omit UGI protein. Analyses for C-E use the same read data set. Data are the average of two technical replicates; error bars represent the standard deviation

We first tested whether the fused UGI domains in our base editor proteins were sufficient to effect meaningful UDG inhibition. To do so, we purified protein variants that lacked the UGI domains (Table S1), complexed these with sgRNAs targeting genomic sites, and transfected the RNPs into HEK293 cells. We found that, for both the Flexible and Precision proteins, the variant without fused UGI domains yielded higher levels of editing with no change in the pattern of editing as compared with the variants containing UGI (Fig. 2A&B, Fig. S3). Further, the removal of the fused UGI domains did not increase the rate of C-to-R substitutions or indels. This suggested that the UGI fusion had negative effects on editing outcomes when the editor was delivered as a recombinant protein, contrary to what has been established based on plasmid overexpression [1, 8].

We then tested co-delivery of UGI protein alongside the CBE protein variants that did not contain the UGI fusion. To maximize the delivery of UGI into the nucleus, we engineered it to contain a 2xNLS tag. For both editors, increasing dosages of UGI protein yielded increasing proportions of substitutions that are C-to-T rather than C-to-R (Fig. 2C). We also observed that the magnitude of this increase was target- and editor- dependent. For example, the proportion of substitutions that were C-to-T at the EMX1-15 site increased from about 74% for RNP alone to about 95% for RNP +15 μg UGI with the Flexible editor, whereas the Precision editor increased this metric from about 82% without UGI to about 96% with 15 μg UGI. At HEKSite2, the proportion of substitutions that were C-to-T increased from about 19% to about 55% for the Flexible editor, while this metric increased from about 22% to about 74% for the Precision editor.

The effect of standalone UGI co-transfection on the absolute rate of C-to-T substitutions was likewise site- and editor-specific (Fig. 2D). For Precision, all four genomic targets yielded increases in the absolute rate of C-to-T editing in a dose-dependent manner. For Flexible, target HEKSite2 exhibited a dose-dependent increase in the absolute rate of C-to-T editing, similar to Precision. For two other targets, EMX1-15 and RNF2, any addition of UGI yielded small increases in C-to-T editing of 20–40% over baseline. For the fourth target, HBB, UGI co-delivery had no effect on the absolute rate of C-to-T editing. The effect of co-transfection with UGI on the rate of indel formation was also editor- and site-specific (Fig. 2E). For both editors, targets EMX1-15 and RNF2 exhibited a dose-dependent reduction in the indel rate with co-transfection of increasing amounts of UGI protein. In contrast, co-transfection of UGI protein increased the rate of indel formation at target HBB for both editors. Indel formation at target HEKSite2 depended on the editor used; indel formation increased for Flexible, while it decreased for Precision. However, co-transfection of UGI protein increased the ratio of C-to-T editing to indel formation for both editors at all targets except HBB (Fig. S4).

**Fig. 3:**
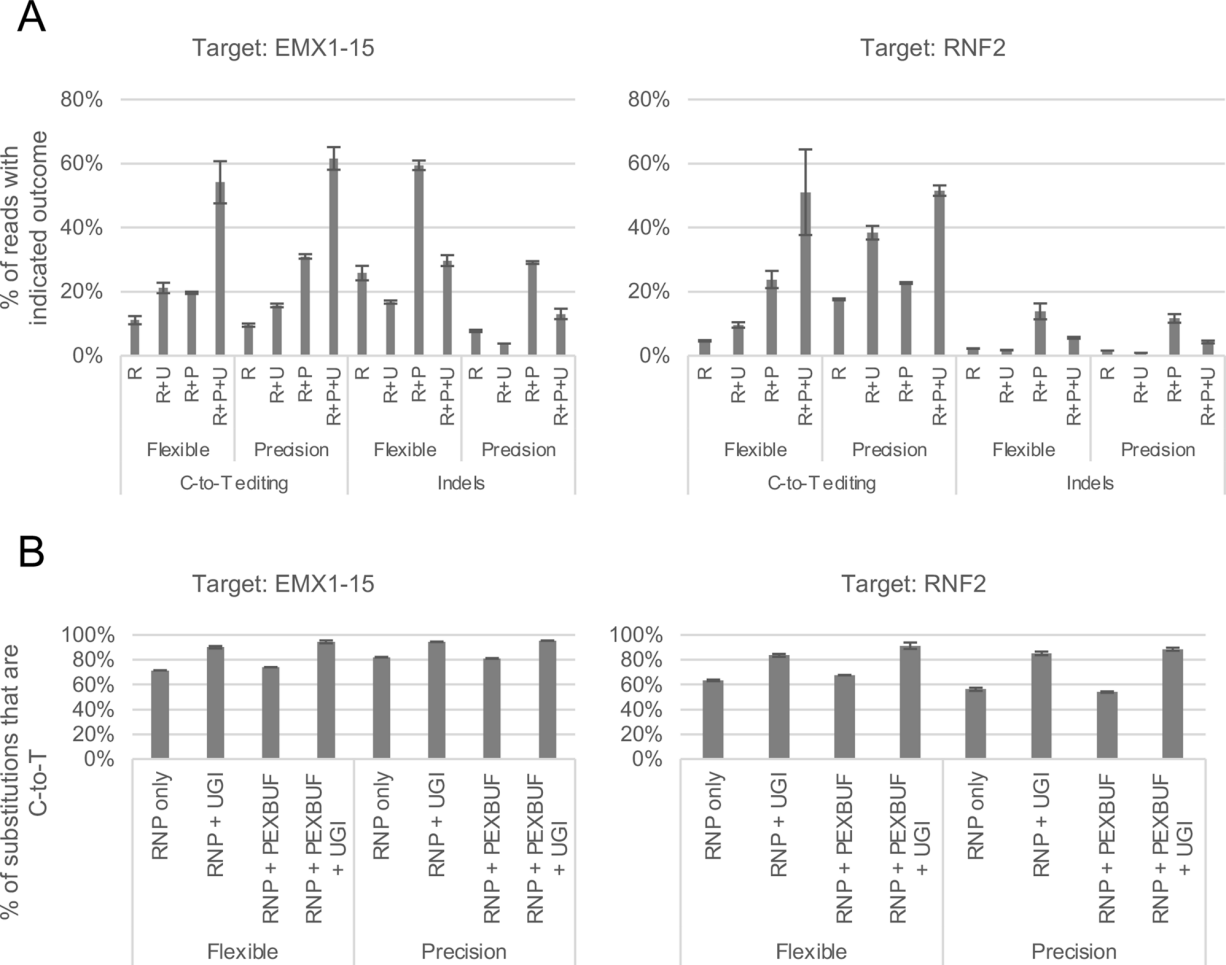
Co-delivery of transfection enhancer PEXBUFF increases overall editing rates. Proteins were purified using gravity column chromatography. Base editor RNPs were delivered into HEK293 cells alone or with 7 μg UGI protein and/or PEXBUFF transfection enhancer. Data are the average of two technical replicates; error bars represent the standard deviation. Reads that contain both substitution and indel are recorded only as indels. See also Fig. S5A) Absolute rates of C-to-T editing and indel formation at genomic targets EMX1-15 and RNF2 for Flexible and Precision editors. R, RNP alone; R+U, RNP + UGI; R+P, RNP + PEXBUFF; R+P+U, RNP + PEXBUFF + UGI. Addition of PEXBUFF increased rates of both outcomes. Use of PEXBUFF and UGI simultaneously further increased rates of C-to-T editing over RNP alone while minimizing indel formation. **B**) Percent of substitutions that are C-to-T at genomic targets EMX1-15 and RNF2 for Flexible and Precision editors. Use of UGI increases the proportion of edits that are C-to-T to a similar degree regardless of the presence/absence of PEXBUFF

We next tested the compatibility of CBE proteins and UGI with the gene editing enhancer PEXBUFF, which is a non-nucleic acid, high molecular weight anionic polymer that boosts rates of gene editing by Cas9 proteins. We transfected HEK293 cells with CBE RNP under 4 conditions: 1) RNP alone; 2) RNP and standalone UGI; 3) RNP and PEXBUFF; and 4) RNP, standalone UGI, and PEXBUFF. As shown in Figs. 3 and S5, we found that co-transfection with PEXBUFF increased overall rates of both C-to-T editing and indel formations. Notably, the proportion of substitutions that were C-to-T remained unchanged, suggesting that PEXBUFF increases deamination without impacting BER. However, the addition of UGI to the transfections resulted in equivalent changes in the proportion of substitutions that are C-to-T, regardless of the presence of PEXBUFF. Thus, PEXBUFF is effective in boosting cytosine base editing and is compatible with the additional benefit conferred by co-transfection with standalone UGI protein.

Having determined their compatibility with standalone UGI and the transfection enhancer, we proceeded to scale up purification of both proteins using an FPLC system. FPLC purified proteins were purer than those purified by gravity filtration as assessed by SDS-PAGE and reliably yielded about 10 mg protein per liter of bacterial culture. Further, FPLC purified material resulted in higher levels of editing in cells (Fig. S6). Subsequent experiments utilized FPLC-purified material

### Editing specificity

Ideally, a genome editing protein will edit only the intended target. In reality, Cas9-based editors exhibit some amount of off-target editing at sites with sequences similar to the target site. This “mismatch” editing can be minimized through careful guide selection; however, this is made more difficult in base editing by the combined requirements for an adjacent PAM sequence and placement of the targeted residue within the editing window. The extended overexpression of editing reagents delivered by plasmid has been shown to result in higher levels of mismatch editing than delivery by RNP [16, 18, 22, 23]. To confirm that our CBEs follow the established pattern, we transfected our base editor proteins into HEK293 cells as either RNPs or plasmids and compared the level of editing, both at the on-target site and at a mismatch site. For RNP transfections, we also evaluated the effect of co-delivery of UGI protein and the transfection enhancer PEXBUFF on specificity.

**Fig. 4:**
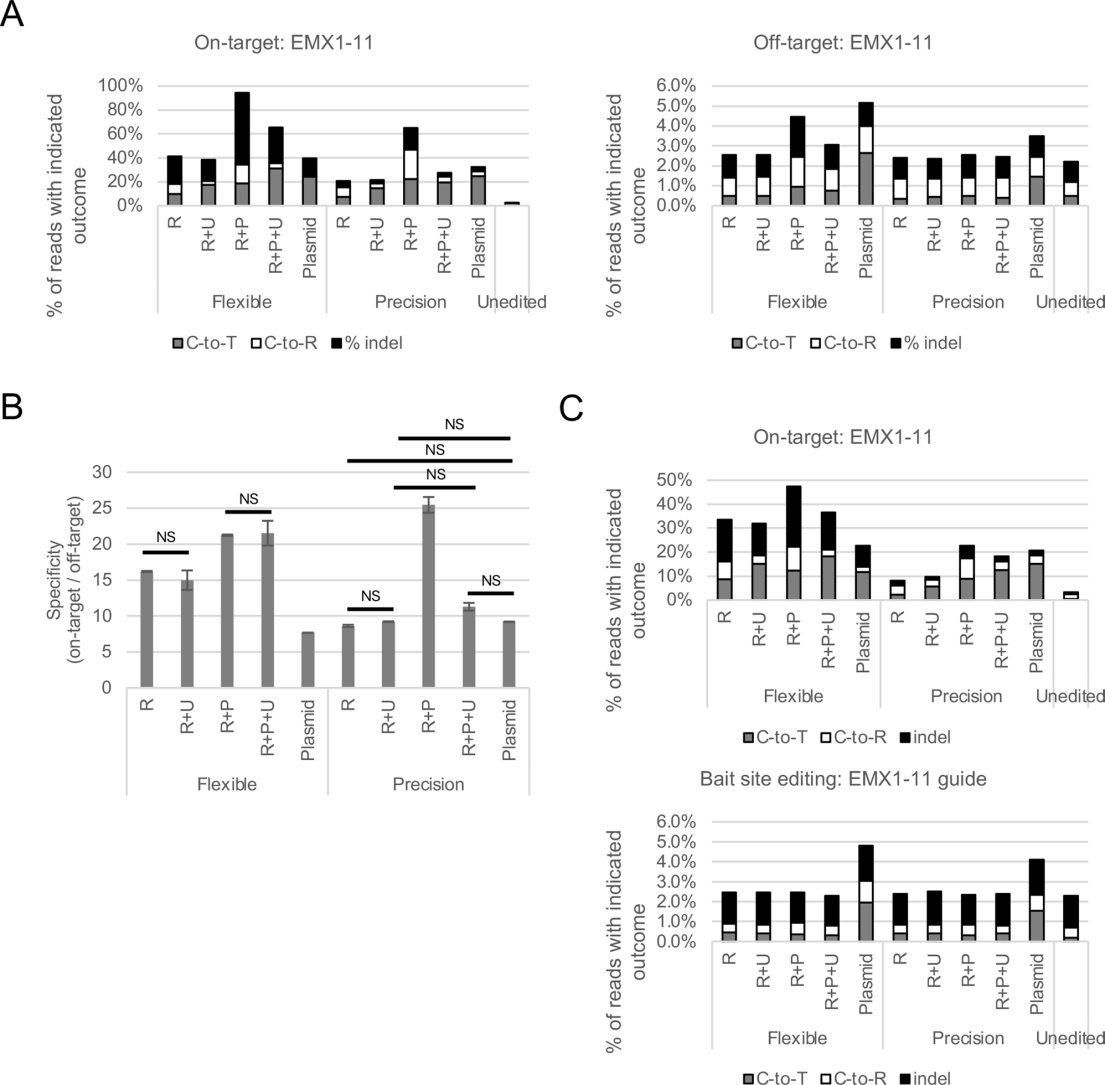
RNP delivery of base editors is more specific than plasmid delivery. Proteins were purified by FPLC. Base editor RNPs were delivered into HEK293 cells alone or with 15 μg UGI protein and/or PEXBUFF transfection enhancer. Data are the average of two technical replicates; error bars represent standard deviation. **A**) Absolute editing rates at the EMX1-11 on-target (left) and mismatch (right) sites. Editing outcomes are indicated by colored stacked bars: gray, C-to-T substitutions; white, C-to-R substitutions; black, indels. **B**) Specificity ratio for editing at the EMX1-11 site. Specificity was calculated by dividing the rate of non-WT reads at the on-target site by the rate of non-WT reads at the mismatch site. Statistics were performed by ANOVA followed by Tukey’s post hoc test; NS indicates *p* > 0.05; all other comparisons are significant (*p* < 0.05). **C**) Absolute editing rates at the EMX1-11 on-target (top) and SaCas9 bait (bottom) sites. Editing outcomes are indicated by colored stacked bars: gray, C-to-T substitutions; white, C-to-R substitutions; black, indels

Results are shown in Fig. 4. For the specificity calculation (Fig. 4B), we define an edit as any sequence changes from wild-type, which encompasses all editing outcomes: C-to-T substitutions, C-to-R substitutions, and indels. For both editors, RNP delivery was no less specific than plasmid delivery under any condition tested, and plasmid delivery yielded the highest absolute rates of editing at the mismatch site. For Precision RNP, editing at the mismatch site was near the level of background (Fig. 4A); therefore, changes in specificity between RNP conditions were driven by changes to the rate of editing at the on-target site. Editing at the mismatch site was higher for Flexible, possibly due to its higher overall editing activity. In addition, transfection with PEXBUFF measurably elevated the rate of mismatch editing; however, on-target editing was boosted considerably more, yielding higher specificity. Co-delivery with UGI protein mitigated the increase in indel formation and C-to-R substitution at both the on-target and mismatch sites.

In addition to mismatch editing, base editors can also edit the genome nonspecifically, in a guide-independent manner [17]. As with mismatch editing, delivery of editing reagents as RNPs has been shown to reduce the rate of these off-target edits [18, 24]. To test whether our CBE proteins could also yield less guide-independent editing than plasmid deliveries, we performed an orthologous R-loop assay as published by Doman et al. [24]. Briefly, nSaCas9 is used to hold open a “bait” region of the genome. Since this region becomes single-stranded, it accumulates a high level of nonspecific editing that can be measured without whole genome sequencing. We transfected HEK293 cells with plasmids expressing a nSaCas9 protein and an SaCas9 guide RNA and either plasmids expressing CBE proteins and base editing guides or CBE RNPs. Plasmid format base editors were delivered simultaneously with the nSaCas9 and guide; RNP format base editors were delivered 24 hours after the nSaCas9 and guide to allow time for expression from the plasmid.

As in the mismatch editing experiment, we found that both plasmid and RNP delivery of base editor resulted in strong editing at the on-target site (Fig. 4C and S7). In contrast, only plasmid delivery of base editor resulted in measurable editing at the bait site. From this, we conclude that our CBE proteins are more specific than plasmid delivery, with regard to both mismatch and genome-wide, nonspecific editing.

As has been observed in other reports [16, 23], we found that we were able to achieve similar or better editing rates with RNP delivery as with plasmid delivery. Notably, the base editor expression plasmids are quite large (8–9 kb), and we observed that some cell types were unable to be edited by plasmid delivery. For example, we observed near-total cell death in A549 cells, but not HEK293 cells, following transfection of base editor plasmids, even after attempts at optimization. Surviving cells were unedited. In contrast, the cells were efficiently edited following RNP delivery of base editor reagents (Fig. S8).

### Characterization of editing window position and sequence context preference

We observed that, at the sites chosen for testing, the editing efficiency was not evenly distributed across the editing window. APOBEC deaminases have known sequence context preferences, with cytosines preceded by thymine (TC dinucleotides) modified more efficiently than other dinucleotide sequences [25, 26]. We hypothesized that our APOBEC3B-based CBE would likely exhibit this characteristic as well, and that this could contribute to the position-to-position disparity in editing observed with the genomic targets. To test this, we developed an in vitro assay to directly measure the editing efficiency at each position of the protospacer, in each dinucleotide context (Fig. S9). Briefly, we designed a set of oligo duplexes with three cytosine residues that tiled across the protospacer, so that each position was covered at least once, in each dinucleotide sequence context. As a “CC” dinucleotide target may possibly be doubly edited to yield a TT outcome, which would be indistinguishable from an edited “TC” dinucleotide target, we included barcodes to enable us to separate these two events bioinformatically. This method allows us to evaluate activity without the potentially confounding variables of chromatin structure and cellular DNA repair pathways.

**Fig. 5:**
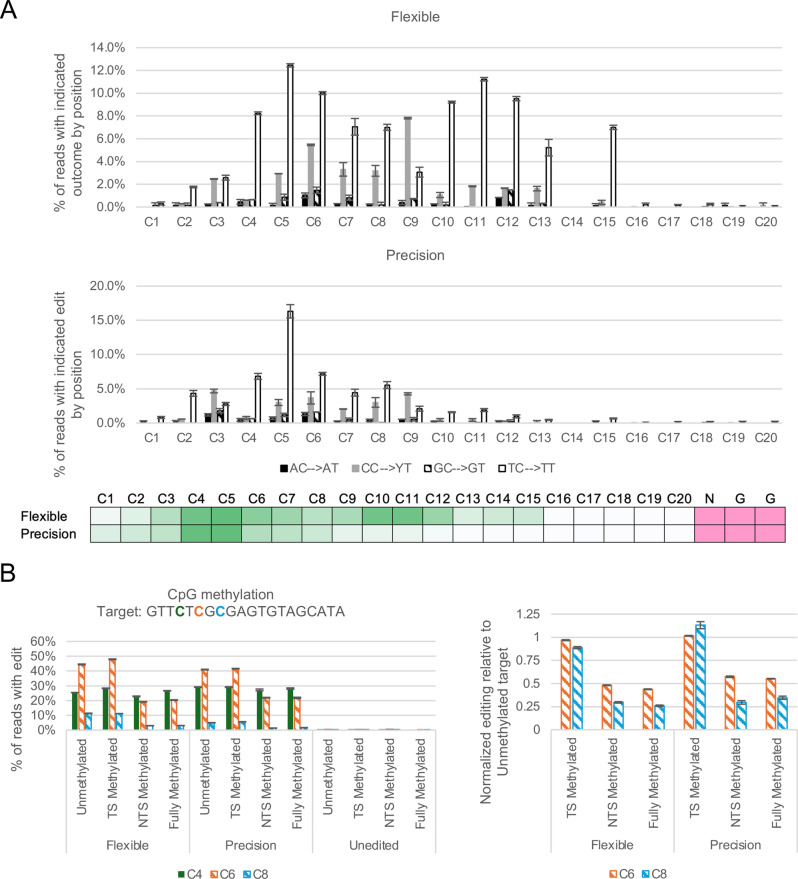
Flexible and Precision exhibit preferences for TC dinucleotides and unmethylated residues. **A**) Editing rate for in vitro reactions to define editing windows and dinucleotide sequence contexts for Flexible (top graph) and Precision (bottom graph). Data are average and standard deviation for two replicates. Editing rates for positions in a CC context include the outcomes CT and TT (YT). Below the plots is a heat map representation of editing rates for each editor, calculated using the editing rates for the TC dinucleotide. **B**) Methylation of cytosines on the non-target strand impedes base editing. The CpG target sequence is given above the charts, with cytosine residues in the editing window bolded and colored as in the plot. Cytosine residues that may be methylated are indicated by hashed bars. Proteins were purified by FLPC. Data for base editor conditions are the average of three technical replicates. Data for unedited controls are the average of two technical replicates, performed on separate days. Error bars represent standard deviation. Left: Absolute C-to-T editing levels of dsDNA oligo targets edited in vitro. Right: Normalized editing relative to the Unmethylated target. First, the rates of editing at each position were normalized to that of the control position (C4). Then, the editing rates for each test condition were normalized to those of the unmethylated target. See also Fig. S10

We found that, as expected from published literature, the CBE proteins had increased activity at “TC” dinucleotide targets (Fig. 5A) relative to other dinucleotide sequence contexts. We further found that “CC” dinucleotide targets were edited more strongly than “AC” or “GC” dinucleotide targets. We note, however, that this preference appears to have a larger effect on the pattern of editing at a target than on the overall level of editing in cells. For example, genomic target HEKSite2 contains only AC and GC dinucleotides but is strongly edited (see Fig. 2C). Within target EMX1-15, we see that the cytosine C11, in an AC dinucleotide context, is weakly edited in relation to the two cytosines residues C9 and C14, which are each in a TC dinucleotide context (see Fig. 1C and S3). Similarly, at target HBB, the cytosine C10 in an AC dinucleotide context is weakly edited compared to the cytosine C8 in a TC dinucleotide context.

We then used the editing data from TC targets to define editing windows for both the basic CBE and the N-terminal SSB variant. We found that the Flexible CBE variant efficiently edited residues from the third position of the protospacer to the 15^th^ position. In contrast, the Precision CBE variant edited from the third to the eighth positions of the protospacer, with a distinct peak at positions four through eight.

### Effect of DNA methylation on editing

One mechanism by which mammals regulate gene expression is through DNA methylation, specifically methylation of the C^5^ position of cytosine residues in CpG dinucleotides. Clusters of CpG dinucleotides, termed CpG islands, are found at 70% of gene promoters, and the incidence of CpG dinucleotides is elevated within exons as well [27]. Promoter methylation results in gene repression, while intragenic methylation has been linked to exon definition [28]. Previous studies have found that many APOBEC deaminases, including APOBEC3B, are sensitive to methylation [29, 30]. We used an in vitro editing assay to determine the effect of methylation on base editing by the Flexible and Precision editors.

Briefly, we designed dsDNA oligonucleotides that harbored CpG or Dcm methylation sites, which were left unmethylated or methylated chemically during oligo synthesis (Fig. S9). Each target also harbored an unmethylated “TC” dinucleotide within the editing window to control for variation in overall editing due to pipetting variance or differences between target DNA preparations. After editing in vitro, we sequenced the oligonucleotide targets by NGS and measured the rate of editing at each cytosine position.

The level of absolute editing of unmethylated control cytosine residues (position 4 in the CpG target and position 3 in the Dcm target, counting from the 5’ end of the sequence) was similar, regardless of methylation status of either strand for both editors (Fig. 5B and S10). This is in line with previous reports showing that Cas9 binding is unaffected by DNA methylation [31]. When the control residues were used to normalize editing, we found that methylation of the edited strand (non-target strand, NTS), but not the sgRNA-binding strand (target strand, TS), compromised editing on both targets and for both editors by 50–75%. The reduction in editing is more pronounced for cytosine residues that are in less favorable editing positions within the target. This suggests that methylated cytosine residues in disfavored sequence contexts or at the edges of the editing window may be particularly susceptible to this effect.

As the effect of methylation on editing efficiency was similar for both Dcm and CpG patterns, methylation inhibition on base editing is likely to be sequence independent. Further, it is likely to impact both in vitro editing and editing in cells. For editing in cells, this has implications for the editing outcomes at methylated residues. C-to-R editing and indel formation is a result of base excision repair activity. However, deamination of methylcytosine yields thymine instead of uracil; therefore, editing of methylated residues is unlikely to result in C-to-R substitutions or indels at that position. Indeed, editing of CpG residues in cells demonstrates an absence of C-to-R substitutions (Fig. S11). The likely methylation state of a target residue should be considered when designing guide RNAs for use in editing experiments. Methylated residues where editing is desired should ideally be placed at favorable positions by guide RNA design to maximize the likelihood of editing. In contrast, methylated residues where editing is not desired may be positioned at the periphery of the editing window in order to further minimize the rate of editing at that undesired position.

### In vitro elucidation of editing patterns for genomic targets

**Fig. 6:**
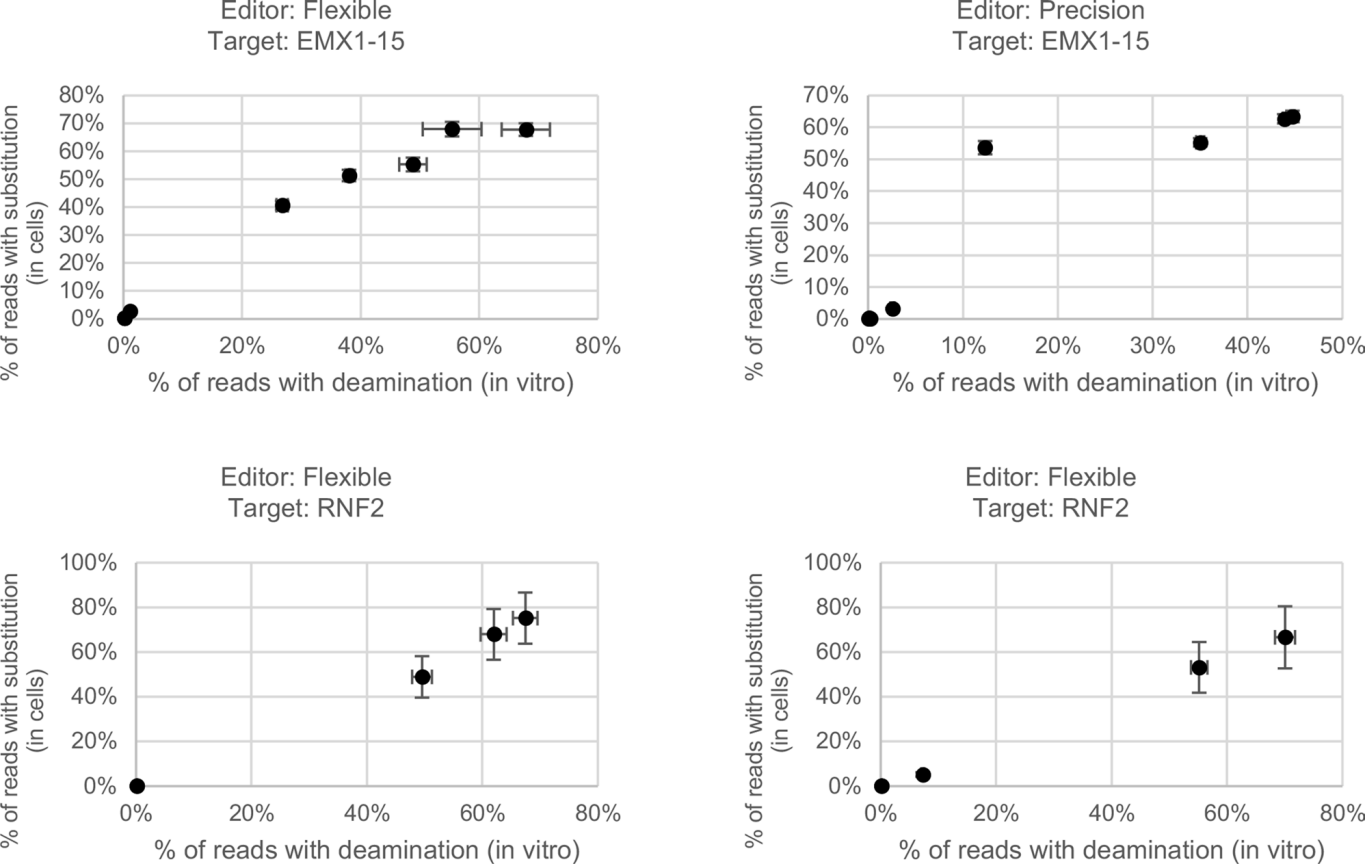
In vitro editing of genomic sites accurately predicts editing patterns in cells. Correlation between editing rates at each cytosine position in cells (y-axis) and in vitro (x-axis). Editing in cells is measured as the sum of all substitutions at each cytosine position, including C-to-R. Each point represents a single cytosine residue. Plots in the left column represent data from editing by Flexible; plots in the right column represent data from editing by precision. Data represent the average of two replicates, and error bars represent the standard deviation. Data for additional sites are given in Fig. S12

We hypothesized that in vitro editing of targets could be used to predict editing patterns in cells. To test this, we amplified genomic target sites from genomic DNA by PCR and subjected the purified PCR products to in vitro editing. In parallel, we subjected HEK293 cells to editing by CBE RNPs. Both in-cell edited targets and in vitro edited targets were subjected to sequencing by NGS and the rate of editing by position was calculated. For in-cell editing, we included all C-to-D (where D is A, G, or T) substitutions to account for any impact of BER. Figs. 6 and S12 show the comparison between editing under these two conditions. For both proteins, there is good correlation between the editing rates in vitro and in the cell. Across the seven sites and both CBE variants, all cytosine residues that were unedited in cells had correspondingly low editing rates in vitro. Of the 33 cytosine residues within the Flexible editing window across the seven sites, 22 (66.7%) were predicted in the correct rank order. 10 (30.0%) residues were mis-predicted by a single rank; four pairs of residues in this category had editing levels in cells that were not statistically significantly different by t-test, so their mis-prediction can be ascribed to chance. Only a single residue (3.0%) was mis-predicted by more than one rank order. Of the 20 cytosine residues within the Precision editing window, all 20 (100%) were predicted with the correct rank order.

This in vitro editing may be used as a preliminary screen for targets or guides to determine whether the intended edit is a likely outcome of an experiment. This would be particularly advantageous in systems with long experimental times, such as plants, where material is precious, such as primary cells, or in embryo editing. In the experiment presented here, we wanted to accurately measure both common and rare editing outcomes. Therefore, we aimed to edit a large number of copies of the targets in accordance with the law of large numbers and chose to use PCR-amplified targets rather than genomic DNA. Because genomes are so large, it is not practical to edit large copy numbers in genomic DNA. However, if identification of the most common outcomes is desired, genomic DNA may be used. This has the added benefit of predicting the effects of potential methylation on editing.

### Editing in additional cell types

**Fig. 7:**
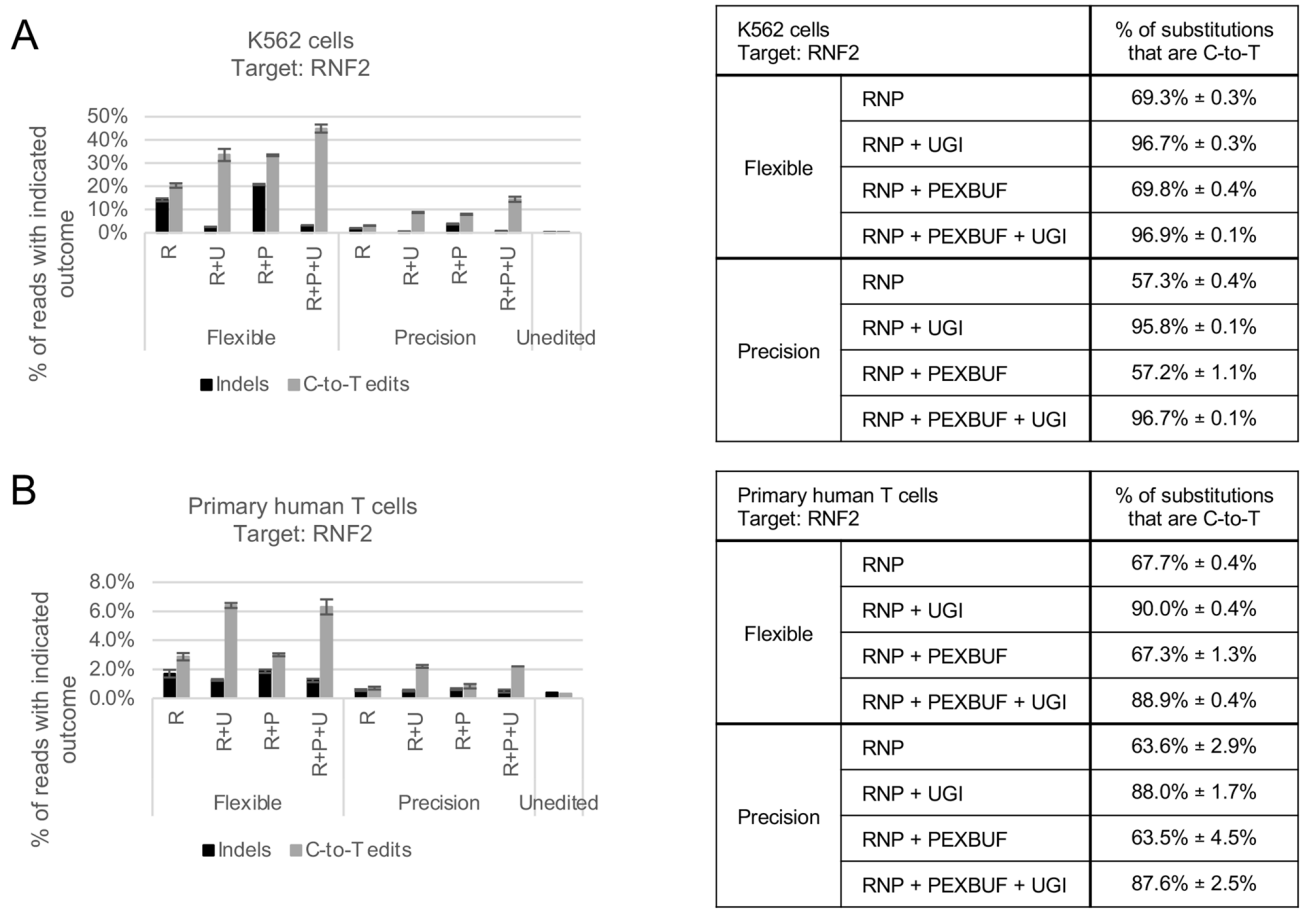
Flexible and Precision are effective in K562 and primary human T cells. Proteins were purified by FPLC. Data are average and standard deviation of three replicates. Left: absolute rates of indel formation (black bars) and C-to-T editing (gray bars) for the genomic target RNF2 in K562 cells (A) and primary human T cells (B). Reads that contain both substitution and indel are recorded only as indels. R, RNP; R+U, RNP + UGI; R+P, RNP + PEXBUFF; R+P+U, RNP + PEXBUFF + UGI. Right: percent of substitutions that are C-to-T for the genomic target RNF2 in K562 cells (A) and primary human T cells (B). See also Figs. S13 and S14

HEK293 cells are an adherent cell line that is particularly amenable to laboratory manipulation. Other cell lines and primary cells may be less easy to transfect or edit. To ensure that our CBE proteins were widely functional, we transfected them into the suspension cell line K562 and into primary human T cells. As shown in Figs. 7A and S13, Flexible and Precision both resulted in efficient editing in K562 cells, and the inclusion of standalone UGI efficiently increased the proportion of C-to-T substitution to above 95% of substitutions. Rates of editing were lower but measurable in primary human T cells (Fig. 7B and S14). The inclusion of PEXBUFF had little impact on the rate of base editing in T cells. However, the inclusion of standalone UGI protein increased both the absolute rate of C-to-T editing and the proportion of substitutions that were C-to-T. For target EMX1-11 (Fig. S14), the addition of UGI also increased the rate of measured indel. When we map the reads to the target, we note that indels occur on edited DNA in a sequence that, following editing, is a poly-T tract. It is unclear whether these indels occur in the cell or are introduced during rounds of PCR and sequencing. Our analysis counts these reads containing both C-to-T substitution and indel only as indels, and despite this, the addition of UGI still results in an increase in the ratio of C-to-T editing to indel formation. Importantly, transfection of T cells with Flexible and Precision CBE variants, regardless of the inclusion of UGI and/or PEXBUFF, did not result in meaningful loss of viability (Fig. S15), as assessed using a luminescence-based assay to measure the amount of ATP, a proxy for metabolically active cells. Thus, Flexible and Precision are able to edit a variety of cell types, including primary cells.

## Conclusion

We have developed two cytosine base editor proteins with complementary features to be commercially available for use in genome editing experiments. The first, Flexible, has a wide editing window (13 nucleotides), allowing for flexible placement of the guide RNA relative to the target residue. The second, Precision, has a narrower window (7 nucleotides), allowing for more discrimination between a target cytosine and any neighboring non-target cytosine residues. Both proteins may be co-transfected with standalone UGI protein to: 1) increase the absolute rate of C-to-T editing; 2) increase the proportion of substitutions that are C-to-T; and/or 3) decrease the rate of indel formation, on a target- and dose-dependent basis. Additionally, both proteins may be co-transfected with the editing enhancer PEXBUFF to increase the overall rate of editing.

Both proteins exhibit a preference for cytosine residues preceded by thymine residues (TC dinucleotides), with lesser but substantial editing of cytosine residues preceded by another cytosine residue (CC dinucleotides), and with further reduced levels of editing at cytosine residues preceded by adenine or guanine residues (AC and GC dinucleotides). In addition, both proteins exhibit a preference for unmethylated cytosine residues; however, editing at methylated cytosine residues is less likely to result in C-to-R substitutions and indels.

Finally, both proteins are more specific than plasmid delivery, in terms of both mismatch editing and guide-independent, genome-wide editing. These proteins can be used in adherent and suspension cell lines, as well as in primary cell types, such as T cells. These proteins provide a readily available tool to facilitate base editing experiments with increased safety and precision over plasmid delivery to laboratories beyond those with the capability to produce CBE proteins in-house.

## Materials & methods

### Protein expression and purification

Plasmids encoding protein of interest (Flexible Base Editor, Precision Base Editor, or UGI) tagged with 6xHis-MBP-HRV3C on the N-terminus in a pET28 plasmid backbone under the T7 promoter were transformed into BL21-AI (DE3) *Escherichia coli* and grown in 250 mL complete auto induction media (Terrific Broth (TB), 1 × 5052 sugars (0.5% glycerol, 0.05% glucose, 0.2% α-lactose), 0.2% arabinose, 2 mM MgSO_4_, 0.2x trace metal solution (Teknova, T1001), 100 ug/mL kanamycin) in a 500 mL baffled Erlenmeyer flask and agitated at 225 RPM at 37 °C to a 600 nM optical density reading of 0.5. The temperature was lowered to 18 °C and fermentation continued for 20 hours. Cells were pelleted at 3200 RCF for 30 minutes. Supernatant was discarded and pellet frozen at -40 °C for at least 12 hours.

Pellets were lysed in 5 mL lysis buffer (50 mM NaH_2_PO_4_, 500 mM NaCl, 300 units Benzonase, 0.5 mg/mL lysozyme, pH 8.0 with 1x SIGMAFAST^TM^ protease inhibitor cocktail) per gram of paste on ice by pipetting and probe sonication. Supernatant was clarified by centrifugation and purified by Ni-NTA resin.

In some experiments, column chromatography was performed by gravity filtration using Ni-NTA His Bind resin (Merck KGaA, Darmstadt, Germany) equilibrated with 50 mM NaH_2_PO_4_, 500 mM NaCl, pH 8. The column was washed with 50 mM NaH_2_PO_4_, 500 mM NaCl, 20 mM imidazole, pH 8 and bound protein eluted with 50 mM NaH_2_PO_4_, 500 mM NaCl, 400 mM imidazole, pH 8. The eluate was dialyzed to 20 mM HEPES, 275 mM NaCl, 10% glycerol, 1 mM DTT, pH 7.8 in a 20 kDa MWCO cellulose membrane dialysis cassette (Thermo Fisher Slide-a-lyzer) according to manufacturer’s instructions and simultaneously proteolyzed with 1 ug HRV3C protease/4 mg total protein (estimated by A280). A second Ni-NTA step was performed to remove tag and uncleaved protein, with the flow-through retained, concentrated to at least 5 mg/mL, dialyzed to 20 mM HEPES, 500 mM NaCl, 10% glycerol, 1 mM DTT, pH 7.8, and stored.

In other experiments, column chromatography was performed for all proteins by FPLC using a HisTrap HP column, with loading, washing, elution, dialysis, and tag cleavage as above. Flexible and Precision proteins then underwent cation exchange chromatography by FPLC using a Cytiva® SP HP cation exchange column equilibrated and washed with 20 mM HEPES, 275 mM NaCl, 1 mM DTT, 10% glycerol pH 7.8. Protein was eluted using a NaCl gradient up to 1 M and fractions from the major elution peaks combined. Flexible and UGI proteins further underwent a second, passive Ni-NTA step using HisTrap HP equilibrated with 20 mM HEPES, 500 mM NaCl, 10% glycerol, 1 mM DTT, 20 mM imidazole pH 7.8. Before loading, the protein solution was adjusted to achieve a final imidazole concentration of 20 mM. Flow-through was collected and the remaining protein of interest was eluted with 20 mM HEPES, 500 mM NaCl, 10% glycerol, 1 mM DTT, 40 mM imidazole pH 7.8. The protein eluates were filtered through a 0.45 μM sterile syringe filter prewet with the formulation buffer (20 mM HEPES, 500 mM NaCl, 50% glycerol, 1 mM DTT, pH 7.8), concentrated to > 5 mg/mL with a regenerated cellulose membrane concentrator (with appropriate MWCO) and dialyzed into formulation buffer. The protein was quantified via BCA, and frozen in aliquots at −80 °C.

### Cell culture

All cells were maintained at 37 °C and 5% CO_2_. HEK293 cells were obtained from ATCC and grown in DMEM supplemented with 10% FBS, 2 mM L-glutamine, 1 mM sodium pyruvate, 0.1 mM non-essential amino acids, and 1x Penicillin-Streptomycin. K562 cells were obtained from ATCC and grown in Iscove’s Modified Dulbecco Medium supplemented with 10% FBS, 2 mM L-Glutamine, and 1X Penicillin-Streptomycin. Human primary CD8+ T cells were purchased from STEMCELL Technologies Inc. and maintained in RPMI 1640 (Thermo Fisher) supplemented with 10% human AB serum (Sigma-Aldrich), 1x GlutaMAX™ (Gibco), 10 ng/mL IL-2 (Gibco), and 50 µM β-mercaptoethanol (Gibco).

### RNP nucleofections

Ribonucleoprotein (RNP) complexes were prepared by incubating for 15 min at room temperature: 40 pmol of CBE protein, 120 pmol sgRNA, and, as appropriate, up to 15 μg of uracil glycosylase inhibitor (UGI) in buffer (20 mM HEPES, 100 mM KCl, 0.5 mM DTT, 0.1 mM EDTA, pH 7.5) for a final volume of 10 μL. RNPs were kept on ice until transfection. Guide sequences are given in Table S2.

HEK293 cells were seeded at 1.67 × 10^4^ cells/cm^2^ of tissue culture surface area two days before transfection. At the time of transfection, cells were trypsinized to obtain a single-cell suspension, washed twice with Hank’s Balanced Salt Solution and resuspended in Nucleofector Solution V (Lonza, Basel, CH) at 2.5 × 10^5^ cells per 100 μL. As appropriate, PEXBUFF (Merck KGaA, Darmstadt, Germany) was added to the cell suspension at 0.5 μL PEXBUFF per 100 μL of suspension. Nucleofection was performed by mixing 100 μL of prepared cell suspension with 10 μL of complexed CBE RNP by pipetting up and down six times before transferring to a cuvette for electroporation using program Q-001 on a Nucleofector 2b machine. Nucleofected cells were immediately transferred to 6-well plates containing 2 mL pre-warmed media per well and grown for 3 days before harvest.

K562 cells were seeded at 2.5 × 10^5^ cells/mL one day before transfection. At the time of transfection, cells were washed twice with Hank’s Balanced Salt Solution and resuspended in Nucleofector Solution V (Lonza, Basel, CH) at 3.5 × 10^5^ cells per 100 μL. As appropriate, PEXBUFF (Merck KGaA, Darmstadt, Germany) was added to the cell suspension at 1.0 μL PEXBUFF per 100 μL of suspension. Nucleofection was performed by mixing 100 μL of prepared cell suspension with 10 μL of complexed CBE RNP before transferring to a cuvette for electroporation using program T-016 on a Nucleofector 2b machine. Nucleofected cells were immediately transferred to 6-well plates containing 2 mL pre-warmed media per well and grown for 3 days before harvest.

Human primary CD8+ T cells were stimulated with T Cell TransAct (Miltenyi Biotec) 3 days prior to nucleofection. Cells were pelleted and resuspended to 2.0 × 10^5^ cells per 100 μL in SF Cell Line Nucleofector Solution. As appropriate, PEXBUFF (Merck KGaA, Darmstadt, Germany) was added to the cell suspension at 1.0 μL PEXBUFF per 100 μL of suspension. Nucleofection was performed by mixing 100 μL of prepared cell suspension with 10 μL of complexed CBE RNP before transferring to a cuvette for electroporation using program DS120 on a Nucleofector 4D machine. Nucleofected cells were immediately transferred to 6-well plates containing 2 mL pre-warmed media per well and grown for 5 days before harvest. Cells were cultured in the presence of TransAct post nucleofection according to manufacturer’s protocol before harvest.

### Plasmid delivery of base editors for mismatch editing

Plasmids were ordered from Azenta that expressed the Flexible CBE (pVax-Flexible) and the Precision CBE (pVax-Precision) under control of the CMV promoter. Plasmids were ordered from Twist Biosciences that expressed guide RNAs for base editors (pTwist-EMX1_11) under control of the U6 promoter.

Plasmids were delivered to HEK293 cells using the *Trans*IT-CRISPR® reagent (Merck KGaA, Darmstadt, Germany). Briefly, cells were plated 1 day before delivery at 1.3 × 10^5^ cells per well of 6-well culture plates. On the day of delivery, 3.0 μg of base editor plasmid and 1.0 μg of guide plasmid were mixed with 12.0 μL of *Trans*IT-CRISPR® reagent in 250 μL of DMEM and incubated at room temperature for 30 min. 250 μL of this mixture was added to the plated cells, and the plates were rocked gently to distribute the mix and incubated for 3 days at 37 °C before harvest.

### NGS library preparation and data analysis

Genomic DNA was isolated from cells by resuspension of harvested cells in 50–75 μL QuickExtract reagent (Lucigen). Suspensions were incubated at 60 °C for 15 m and 95 °C for 15 m. This material was used directly in PCR reactions without further cleanup.

Genomic regions targeted by the CBE were amplified by PCR using JumpStart^TM^ Taq ReadyMix^TM^ reagent (Merck KGaA, Darmstadt, Germany) and the following cycling conditions: 94°C/2 m; 25 cycles of 94°C/30s, 62°C/30s, 72°C/45s; 72°C/5 m. Primers are listed in Table S2. PCR products underwent a second round of amplification using Illumina index primers and JumpStart^TM^ Taq ReadyMix^TM^ reagent and the following conditions: 95°C/3 m; 9 cycles of 95°C/30s, 55°C/30s, 72°C/30s; 72°C/5 m. Indexed PCR products were purified by Select-a-Size DNA Clean & Concentrator MagBeads (Zymo, Irvine, CA), using 1.2x beads by volume, quantified by PicoGreen (Thermo Fisher, Waltham, MA), and pooled according to DNA content. Pools were diluted to 4 nM. Sequencing was performed on an Illumina MiSeq instrument using a 300-cycle kit to obtain single-end reads. FASTQ files for each sample were analyzed using CRIS.py [32] or a custom analysis script (Supplemental Text).

### Orthologous R-loop assay

Plasmids were ordered from Azenta that expressed nSaCas9 (pVax-nSaCas9) under control of the CMV promoter. Plasmids were ordered from Twist Biosciences that expressed guide RNAs for SaCas9 (pTwist-Doman5) under control of the U6 promoter. Base editor plasmids were as described above.

RNP complexes were prepared by incubating for 15 min at room temperature: 40 pmol of CBE protein, 120 pmol sgRNA, and, as appropriate, 15 μg of UGI in buffer (20 mM HEPES, 100 mM KCl, 0.5 mM DTT, 0.1 mM EDTA, pH 7.5) for a final volume of 10 μL. RNPs were kept on ice until transfection.

On Day 1 of the experiment, HEK293 cells were transfected with plasmids at 2.5 × 10^5^ cells/transfection using the Lonza Nucleofector 2b. Each transfection contained 2.0 μg of base editor plasmid and 1.0 μg of EMX1-11 guide plasmid. Some transfections also contained 2.0 μg of nSaCas9 plasmid and 1.0 μg of Doman5 guide plasmid. Cells were grown for 3 days in 6-well plates before harvest.

Separately, 8.0 × 10^6^ cells were nucleofected in batches with a total of 64.0 μg of nSaCas9 plasmid and 32.0 μg of Doman5 guide plasmid. Cells were pooled, split between 2× T150 flasks, and grown overnight. On Day 2, the cells were again pooled and nucleofected with RNPs at 2.5 × 10^5^ cells per sample. As appropriate, PEXBUFF was added at 0.5 μL per 100 μL of cell suspension. In parallel, RNPs (with and without PEXBUFF) were nucleofected into cells that had not received any plasmid components at the same ratio of cells to RNP. RNP nucelofections were grown for 3 days in 6-well plates before harvest.

Cell harvest, library preparation, sequencing, and data analysis were performed as above.

### In vitro editing window and sequence specificity assay

Double-stranded DNA targets were constructed by cloning annealed oligos into a plasmid backbone. Insert sequences are given in Table S3. Each insert contains a unique 6nt barcode, a target sequence containing three cytosine residues on the non-targeted strand, and a 5’-TGG-3’ PAM. Plasmid sequences were confirmed by sequencing before experiments were performed. Plasmids were pooled such that the NC dinucleotide frequency was equal for each value of N (where *N* = any base) and the frequency of cytosine at each position was equal.

Synthetic crRNAs were designed to target the pool of dsDNAs and ordered from IDT (Integrated DNA Technologies, Coralville, IA). crRNAs targeting dsDNAs harboring VC dinucleotides were synthesized with degenerate bases at the relevant positions. crRNAs were pooled such that the NC dinucleotide frequency was equal for each value of N and the frequency of cytosine at each position was equal. The crRNA pool was complexed with an equimolar amount of trRNA. RNPs were assembled by mixing 20 pmol of base editor protein with 20 pmol of pooled crRNA:trRNA complex and incubating at RT for 15 min.

Reactions were performed in triplicate and each contained 20 fmol of target pool and 6 pmol of RNP in a 20 μL reaction in assay buffer (20 mM Tris, 100 mM NaCl, 5 mM MgCl_2_). Reactions were incubated at 37 °C for 10 min and quenched at 80 °C for 15 min. The target region was amplified by PCR using JumpStart^TM^ Taq ReadyMix^TM^ reagent (Merck KGaA, Darmstadt, Germany) and the following cycling conditions: 94 °C/2 m; 25 cycles of 94 °C/30s, 58 °C/30s, 72 °C/45s; 72 °C/2 m. PCR products underwent a second round of amplification using Illumina (San Diego, CA) index primers and JumpStart^TM^ Taq ReadyMix^TM^ reagent and the following conditions: 95 °C/3 m; 9 cycles of 95 °C/30s, 55 °C/30s, 72 °C/30s; 72 °C/5 m. Indexed PCR products were quantified by PicoGreen (Thermo Fisher, Waltham, MA), purified by Select-a-Size DNA Clean & Concentrator MagBeads (Zymo, Irvine, CA) using 1.2x beads by volume, and pooled according to DNA content. Pools were diluted to 4 nM. Sequencing was performed on an Illumina MiSeq instrument using a 300-cycle kit to obtain single-end reads. FASTQ files for each sample were analyzed using a custom analysis script and the CRIS.py software package.

### In vitro editing outcome prediction

Genomic targets were amplified as detailed for NGS library preparation and purified using Select-a-Size DNA Clean & Concentrator MagBeads (Zymo, Irvine, CA), using 1.2x beads by volume, and quantified by Qubit HS assay (Thermo Fisher, Waltham, MA). RNPs were assembled by incubating 50 pmol of base editor protein with 50 pmol guide in buffer (20 mM Tris, 100 mM NaCl, 5 mM MgCl_2_) at 6 pmol per 10 μL and incubating at RT for 10 min. Purified amplicon targets and RNPs were mixed in 20 μL reactions containing 6 pmol RNP and 100 fmol target DNA, incubated at 37 °C for 45 min, and quenched at 80 °C for 15 min. Edited PCR products were amplified using Illumina index primers and JumpStart^TM^ Taq ReadyMix^TM^ reagent and the following conditions: 95°C/3 m; 9 cycles of 95°C/30s, 55°C/30s, 72°C/30s; 72°C/5 m. Indexed PCR products were purified by Select-a-Size DNA Clean & Concentrator MagBeads (Zymo, Irvine, CA), using 1.2x beads by volume, quantified by PicoGreen (Thermo Fisher, Waltham, MA), and pooled according to DNA content. Pools were diluted to 4 nM. Sequencing was performed on an Illumina MiSeq instrument and FASTQ files analyzed using the CRIS.py software package. Briefly, we calculated the fraction of each dinucleotide at each position that was edited for each replicate. We then subtracted the “editing rate” of the unedited control from each replicate for each data point. As some editing rates were at background, if the calculated rate of editing for the control was above that of the sample, we set the editing rate for that point to zero.

### In vitro methylation sensitivity assay

Pairs of DNA oligonucleotides were ordered from Merck KGaA, Darmstadt, Germany that, when annealed, form perfectly complementary dsDNA targets. Sequences of target oligos, guides, and primers can be found in Table S4. Oligos were ordered with and without cytosine methylation in sequences characteristic of Dcm and CpG methylation. Oligos were annealed at 5 μM in a buffer containing 10 mM Tris, 50 mM NaCl, and 1 mM EDTA. Synthetic sgRNAs were ordered from Merck KGaA, Darmstadt, Germany to target the dsDNA oligos. RNPs were assembled by incubating 20 pmol of base editor protein with 20 pmol guide in buffer (20 mM Tris, 100 mM NaCl, 5 mM MgCl_2_) at 6 pmol per 10 μL and incubating at RT for 10 min. Annealed targets and RNPs were mixed in 20 μL reactions containing 6 pmol RNP and 100 fmol target DNA, incubated at 37 °C for 1 hr, and quenched at 80 °C for 15 min. Targets were amplified by PCR using JumpStart^TM^ Taq ReadyMix^TM^ reagent (Merck KGaA, Darmstadt, Germany) and the following cycling conditions: 94°C/2 m; 25 cycles of 94°C/30s, 58°C/30s, 72°C/45s; 72°C/5 m. PCR products underwent a second round of amplification using Illumina index primers and JumpStart^TM^ Taq ReadyMix^TM^ reagent and the following conditions: 95°C/3 m; 9 cycles of 95°C/30s, 55°C/30s, 72°C/30s; 72°C/5 m. Indexed PCR products were purified by Select-a-Size DNA Clean & Concentrator MagBeads (Zymo, Irvine, CA), using 1.2x beads by volume, quantified by PicoGreen (Thermo Fisher, Waltham, MA), and pooled according to DNA content. Pools were diluted to 4 nM. Sequencing was performed on an Illumina MiSeq instrument and FASTQ files analyzed using the custom base editor analysis script.

### Cell viability assay

Cell viability was tested with the CellTiter-Glo® 2.0 Assay (Promega, Madison, WI). An opaque-walled 96-well plate was prepared with 50 μL of each well from the 48-plate after thoroughly mixing and left at room temperature for 30 minutes. 50 μL of room temperature CellTiter-Glo® 2.0 Reagent was then added to each well and mixed on an orbital shaker for 10 minutes. Luminescence was then read on a SpectraMax iD3 (Danaher, Washington, DC).

## Electronic supplementary material

Below is the link to the electronic supplementary material.

Supplementary Material 1

## Data Availability

Lead contact: Requests for further information and resources should be directed to the lead contact, Erin Brettmann (erin.brettmann@milliporesigma.com). Materials availability: All unique/stable reagents generated in this study are available from the commercial provider as catalog products CAS9CBEFLX and CAS9CBEPRC.

## Code availability

1. Amplicon sequencing reads have been deposited in SRA and are publicly available as of the date of publication as BioProject accession PRJNA1232714. 2. Custom code for the NGS data analysis is provided in the supplemental text. 3. Any additional information required to reanalyze the data reported in this paper is available from the lead contact upon request.
