# Supplementary material for: Cytosine base editor-DNA binding domain fusions for editing window modulation in the RNP format
Source: BMC Biotechnol. 2025 Aug 29;25:92. doi: 10.1186/s12896-025-01020-1 (PMC12395855; doi:10.1186/s12896-025-01020-1)
Supplement: Supplementary file 1 — Supplementary Material 1 [file 12896_2025_1020_MOESM1_ESM.docx]

**Supplemental Text**

Custom script for base editing read data analysis

Dependencies:

Bowtie2

Samtools

Bam-readcount

GNU sed

Required are:

- An indexed fasta file with your amplicon reference sequence
- A bowtie2 reference index for your fasta reference sequence file
- A bed file defining positions containing cytosine residues of interest within your fasta reference sequence file

#!/bin/sh

#target being tested

TARGET=

#directory containing fastq.gz files

DIR=

for FILE in ${DIR}/*.fastq.gz

#align reads to amplicon with bowtie2

do echo $FILE >> ${DIR}/${TARGET}_bowtieStats.txt

bowtie2 -x Targets -U $FILE --local -S ${FILE%.fastq.gz}_align.sam 2>> ${DIR}/${TARGET}_bowtieStats.txt

samtools view -b -o ${FILE%.fastq.gz}_align.bam ${FILE%.fastq.gz}_align.sam

samtools sort -o ${FILE%.fastq.gz}_align_sort.bam ${FILE%.fastq.gz}_align.bam

samtools index ${FILE%.fastq.gz}_align_sort.bam

#get base coverage at positions of interest

bam-readcount -l ${TARGET}_loc.bed -f Targets.fa ${FILE%.fastq.gz}_align_sort.bam ${TARGET} > ${FILE%.fastq.gz}_${TARGET}_bamReadcount

#get only C & T bases

awk '{print $1","$2","$3","$4","$7","$9}' ${FILE%.fastq.gz}_${TARGET}_bamReadcount > ${FILE%.fastq.gz}_${TARGET}_bamReadcount_T-filtered

#get only read counts, calculate editing, & output csv for C --> T editing

gsed 's/:/,/g' ${FILE%.fastq.gz}_${TARGET}_bamReadcount_T-filtered > ${FILE%.fastq.gz}_${TARGET}_bamReadcount_T-filtered_comma

awk -F',' '{print $1","$2","$3","$4","$6","$20","$6/$4","$20/$4}' ${FILE%.fastq.gz}_${TARGET}_bamReadcount_T-filtered_comma > ${FILE%.fastq.gz}_${TARGET}_C-T_readcounts.csv

#get only C & G bases

awk '{print $1","$2","$3","$4","$7","$8}' ${FILE%.fastq.gz}_${TARGET}_bamReadcount > ${FILE%.fastq.gz}_${TARGET}_bamReadcount_G-filtered

#get only read counts, calculate editing, & output csv for C --> G editing

gsed 's/:/,/g' ${FILE%.fastq.gz}_${TARGET}_bamReadcount_G-filtered > ${FILE%.fastq.gz}_${TARGET}_bamReadcount_G-filtered_comma

awk -F',' '{print $1","$2","$3","$4","$6","$20","$6/$4","$20/$4}' ${FILE%.fastq.gz}_${TARGET}_bamReadcount_G-filtered_comma > ${FILE%.fastq.gz}_${TARGET}_C-G_readcounts.csv

#get only C & A bases

awk '{print $1","$2","$3","$4","$6","$7}' ${FILE%.fastq.gz}_${TARGET}_bamReadcount > ${FILE%.fastq.gz}_${TARGET}_bamReadcount_A-filtered

#get only read counts, calculate editing, & output csv for C --> A editing

gsed 's/:/,/g' ${FILE%.fastq.gz}_${TARGET}_bamReadcount_A-filtered > ${FILE%.fastq.gz}_${TARGET}_bamReadcount_A-filtered_comma

awk -F',' '{print $1","$2","$3","$4","$6","$20","$6/$4","$20/$4}' ${FILE%.fastq.gz}_${TARGET}_bamReadcount_A-filtered_comma > ${FILE%.fastq.gz}_${TARGET}_C-A_readcounts.csv

#remove intermediate files

rm ${FILE%.fastq.gz}_align.sam

rm ${FILE%.fastq.gz}_align.bam

rm ${FILE%.fastq.gz}_${TARGET}_bamReadcount_T-filtered_comma

rm ${FILE%.fastq.gz}_${TARGET}_bamReadcount_G-filtered_comma

rm ${FILE%.fastq.gz}_${TARGET}_bamReadcount_A-filtered_comma

rm ${FILE%.fastq.gz}_${TARGET}_bamReadcount_G-filtered

rm ${FILE%.fastq.gz}_${TARGET}_bamReadcount_T-filtered

rm ${FILE%.fastq.gz}_${TARGET}_bamReadcount_A-filtered

done

#assemble samples into a single file

for file in ${DIR}/*${TARGET}*C-T_readcounts.csv

do

echo ${file} >> ${DIR}/${TARGET}_T-sampleFiles

awk -F',' '{print $2}' $file | paste -d, -s - > ${DIR}/${TARGET}_sites.csv

awk -F',' '{print $8}' $file | paste -s -d, - >> ${DIR}/${TARGET}_allSamples_T-edited.csv

done

for file in ${DIR}/*${TARGET}*C-G_readcounts.csv

do

echo ${file} >> ${DIR}/${TARGET}_G-sampleFiles

awk -F',' '{print $8}' $file | paste -s -d, - >> ${DIR}/${TARGET}_allSamples_G-edited.csv

done

for file in ${DIR}/*${TARGET}*C-A_readcounts.csv

do

echo ${file} >> ${DIR}/${TARGET}_A-sampleFiles

awk -F',' '{print $7}' $file | paste -s -d, - >> ${DIR}/${TARGET}_allSamples_A-edited.csv

done

cat ${DIR}/${TARGET}_sites.csv ${DIR}/${TARGET}_allSamples_T-edited.csv > ${DIR}/${TARGET}_sites_allSamples_T-edited.csv

gsed -i '1i\\' ${DIR}/${TARGET}_T-sampleFiles

paste -d, ${DIR}/${TARGET}_T-sampleFiles ${DIR}/${TARGET}_sites_allSamples_T-edited.csv > ${DIR}/${TARGET}_C-T_editing_results.csv

cat ${DIR}/${TARGET}_sites.csv ${DIR}/${TARGET}_allSamples_G-edited.csv > ${DIR}/${TARGET}_sites_allSamples_G-edited.csv

gsed -i '1i\\' ${DIR}/${TARGET}_G-sampleFiles

paste -d, ${DIR}/${TARGET}_G-sampleFiles ${DIR}/${TARGET}_sites_allSamples_G-edited.csv > ${DIR}/${TARGET}_C-G_editing_results.csv

cat ${DIR}/${TARGET}_sites.csv ${DIR}/${TARGET}_allSamples_A-edited.csv > ${DIR}/${TARGET}_sites_allSamples_A-edited.csv

gsed -i '1i\\' ${DIR}/${TARGET}_A-sampleFiles

paste -d, ${DIR}/${TARGET}_A-sampleFiles ${DIR}/${TARGET}_sites_allSamples_A-edited.csv > ${DIR}/${TARGET}_C-A_editing_results.csv

#remove intermediate files

rm ${DIR}/${TARGET}_T-sampleFiles

rm ${DIR}/${TARGET}_G-sampleFiles

rm ${DIR}/${TARGET}_A-sampleFiles

rm ${DIR}/${TARGET}_sites.csv

rm ${DIR}/${TARGET}_allSamples_T-edited.csv

rm ${DIR}/${TARGET}_allSamples_G-edited.csv

rm ${DIR}/${TARGET}_allSamples_A-edited.csv

rm ${DIR}/${TARGET}_sites_allSamples_T-edited.csv

rm ${DIR}/${TARGET}_sites_allSamples_G-edited.csv

rm ${DIR}/${TARGET}_sites_allSamples_A-edited.csv

#organize files

cd ${DIR}

mkdir NewDir

for File in *{TARGET}*

do mv $File NewDir/$File

done

Table S1: Amino acid sequences of proteins. Single stranded DNA binding protein domains are underlined. Deaminase domains are bolded. Uracil glycosylase inhibitor domains are italicized.

| Protein | Amino acid sequence |
| --- | --- |
| **A3B**-nCas9-*2U* | GPGS**EILRYLMDPDTFTFNFNNDPLVLRRRQTYLCYEVERLDNGTWVLMDQHMGFLCNEAKNLLCGFYGRHAELRFLDLVPSLQLDPAQIYRVTWFISWSPCFSWGCAGEVRAFLQENTHVRLRIFAARIYDYDPLYKEALQMLRDAGAQVSIMTYDEFEYCWDTFVYRQGCPFQPWDGLEEHSQALSGRLRAILQ**ASSGGSSGGSSGSETPGTSESATPESSGGSSGGSPAAKRVKLDGGGGSPKKKRKVTGMDKKYSIGLAIGTNSVGWAVITDEYKVPSKKFKVLGNTDRHSIKKNLIGALLFDSGETAEATRLKRTARRRYTRRKNRICYLQEIFSNEMAKVDDSFFHRLEESFLVEEDKKHERHPIFGNIVDEVAYHEKYPTIYHLRKKLVDSTDKADLRLIYLALAHMIKFRGHFLIEGDLNPDNSDVDKLFIQLVQTYNQLFEENPINASGVDAKAILSARLSKSRRLENLIAQLPGEKKNGLFGNLIALSLGLTPNFKSNFDLAEDAKLQLSKDTYDDDLDNLLAQIGDQYADLFLAAKNLSDAILLSDILRVNTEITKAPLSASMIKRYDEHHQDLTLLKALVRQQLPEKYKEIFFDQSKNGYAGYIDGGASQEEFYKFIKPILEKMDGTEELLVKLNREDLLRKQRTFDNGSIPHQIHLGELHAILRRQEDFYPFLKDNREKIEKILTFRIPYYVGPLARGNSRFAWMTRKSEETITPWNFEEVVDKGASAQSFIERMTNFDKNLPNEKVLPKHSLLYEYFTVYNELTKVKYVTEGMRKPAFLSGEQKKAIVDLLFKTNRKVTVKQLKEDYFKKIECFDSVEISGVEDRFNASLGTYHDLLKIIKDKDFLDNEENEDILEDIVLTLTLFEDREMIEERLKTYAHLFDDKVMKQLKRRRYTGWGRLSRKLINGIRDKQSGKTILDFLKSDGFANRNFMQLIHDDSLTFKEDIQKAQVSGQGDSLHEHIANLAGSPAIKKGILQTVKVVDELVKVMGRHKPENIVIEMARENQTTQKGQKNSRERMKRIEEGIKELGSQILKEHPVENTQLQNEKLYLYYLQNGRDMYVDQELDINRLSDYDVDHIVPQSFLKDDSIDNKVLTRSDKNRGKSDNVPSEEVVKKMKNYWRQLLNAKLITQRKFDNLTKAERGGLSELDKAGFIKRQLVETRQITKHVAQILDSRMNTKYDENDKLIREVKVITLKSKLVSDFRKDFQFYKVREINNYHHAHDAYLNAVVGTALIKKYPKLESEFVYGDYKVYDVRKMIAKSEQEIGKATAKYFFYSNIMNFFKTEITLANGEIRKRPLIETNGETGEIVWDKGRDFATVRKVLSMPQVNIVKKTEVQTGGFSKESILPKRNSDKLIARKKDWDPKKYGGFDSPTVAYSVLVVAKVEKGKSKKLKSVKELLGITIMERSSFEKNPIDFLEAKGYKEVKKDLIIKLPKYSLFELENGRKRMLASAGELQKGNELALPSKYVNFLYLASHYEKLKGSPEDNEQKQLFVEQHKHYLDEIIEQISEFSKRVILADANLDKVLSAYNKHRDKPIREQAENIIHLFTLTNLGAPAAFKYFDTTIDRKRYTSTKEVLDATLIHQSITGLYETRIDLSQLGGDEFSGGSGGSGGS*TNLSDIIEKETGKQLVIQESILMLPEEVEEVIGNKPESDILVHTAYDESTDENVMLLTSDAPEYKPWALVIQDSNGENKIKML*SGGSGGSGGS*TNLSDIIEKETGKQLVIQESILMLPEEVEEVIGNKPESDILVHTAYDESTDENVMLLTSDAPEYKPWALVIQDSNGENKIKML*SGGSRSPKKKRKVGGGGSPKKKRKV |
| T4-**A3B**-nCas9-*2U* | GPGSKGFSSEDKGEWKLKLDNAGNGQAVIRFLPSKNDEQAPFAILVNHGFKKNGKWYIETCSSTHGDYDSCPVCQYISKNDLYNTDNKEYSLVKRKTSYWANILVVKDPAAPENEGKVFKYRFGKKIWDKINAMIAVDVEMGETPVDVTCPWEGANFVLKVKQVSGFSNYDESKFLNQSAIPNIDDESFQKELFEQMVDLSEMTSKDKFKSFEELNTKFGQVMGTAVMGGAAATAAKKAGGGGSVD**EILRYLMDPDTFTFNFNNDPLVLRRRQTYLCYEVERLDNGTWVLMDQHMGFLCNEAKNLLCGFYGRHAELRFLDLVPSLQLDPAQIYRVTWFISWSPCFSWGCAGEVRAFLQENTHVRLRIFAARIYDYDPLYKEALQMLRDAGAQVSIMTYDEFEYCWDTFVYRQGCPFQPWDGLEEHSQALSGRLRAILQ**ASSGGSSGGSSGSETPGTSESATPESSGGSSGGSPAAKRVKLDGGGGSPKKKRKVTGMDKKYSIGLAIGTNSVGWAVITDEYKVPSKKFKVLGNTDRHSIKKNLIGALLFDSGETAEATRLKRTARRRYTRRKNRICYLQEIFSNEMAKVDDSFFHRLEESFLVEEDKKHERHPIFGNIVDEVAYHEKYPTIYHLRKKLVDSTDKADLRLIYLALAHMIKFRGHFLIEGDLNPDNSDVDKLFIQLVQTYNQLFEENPINASGVDAKAILSARLSKSRRLENLIAQLPGEKKNGLFGNLIALSLGLTPNFKSNFDLAEDAKLQLSKDTYDDDLDNLLAQIGDQYADLFLAAKNLSDAILLSDILRVNTEITKAPLSASMIKRYDEHHQDLTLLKALVRQQLPEKYKEIFFDQSKNGYAGYIDGGASQEEFYKFIKPILEKMDGTEELLVKLNREDLLRKQRTFDNGSIPHQIHLGELHAILRRQEDFYPFLKDNREKIEKILTFRIPYYVGPLARGNSRFAWMTRKSEETITPWNFEEVVDKGASAQSFIERMTNFDKNLPNEKVLPKHSLLYEYFTVYNELTKVKYVTEGMRKPAFLSGEQKKAIVDLLFKTNRKVTVKQLKEDYFKKIECFDSVEISGVEDRFNASLGTYHDLLKIIKDKDFLDNEENEDILEDIVLTLTLFEDREMIEERLKTYAHLFDDKVMKQLKRRRYTGWGRLSRKLINGIRDKQSGKTILDFLKSDGFANRNFMQLIHDDSLTFKEDIQKAQVSGQGDSLHEHIANLAGSPAIKKGILQTVKVVDELVKVMGRHKPENIVIEMARENQTTQKGQKNSRERMKRIEEGIKELGSQILKEHPVENTQLQNEKLYLYYLQNGRDMYVDQELDINRLSDYDVDHIVPQSFLKDDSIDNKVLTRSDKNRGKSDNVPSEEVVKKMKNYWRQLLNAKLITQRKFDNLTKAERGGLSELDKAGFIKRQLVETRQITKHVAQILDSRMNTKYDENDKLIREVKVITLKSKLVSDFRKDFQFYKVREINNYHHAHDAYLNAVVGTALIKKYPKLESEFVYGDYKVYDVRKMIAKSEQEIGKATAKYFFYSNIMNFFKTEITLANGEIRKRPLIETNGETGEIVWDKGRDFATVRKVLSMPQVNIVKKTEVQTGGFSKESILPKRNSDKLIARKKDWDPKKYGGFDSPTVAYSVLVVAKVEKGKSKKLKSVKELLGITIMERSSFEKNPIDFLEAKGYKEVKKDLIIKLPKYSLFELENGRKRMLASAGELQKGNELALPSKYVNFLYLASHYEKLKGSPEDNEQKQLFVEQHKHYLDEIIEQISEFSKRVILADANLDKVLSAYNKHRDKPIREQAENIIHLFTLTNLGAPAAFKYFDTTIDRKRYTSTKEVLDATLIHQSITGLYETRIDLSQLGGDEFSGGSGGSGGS*TNLSDIIEKETGKQLVIQESILMLPEEVEEVIGNKPESDILVHTAYDESTDENVMLLTSDAPEYKPWALVIQDSNGENKIKML*SGGSGGSGGS*TNLSDIIEKETGKQLVIQESILMLPEEVEEVIGNKPESDILVHTAYDESTDENVMLLTSDAPEYKPWALVIQDSNGENKIKML*SGGSRSPKKKRKVGGGGSPKKKRKV |
| T7-**A3B**-nCas9-*2U* | GPGSMAKKIFTSALGTAEPYAYIAKPDYGNEERGFGNPRGVYKVDLTIPNKDPRCQRMVDEIVKCHEEAYAAAVEEYEANPPAVARGKKPLKPYEGDMPFFDNGDGTTTFKFKCYASFQDKKTKETKHINLVVVDSKGKKMEDVPIIGGGSKLKVKYSLVPYKWNTAVGASVKLQLESVMLVELATFGGGEDDWADEVEENGYVASGSAKASKPRGGGGSVD**EILRYLMDPDTFTFNFNNDPLVLRRRQTYLCYEVERLDNGTWVLMDQHMGFLCNEAKNLLCGFYGRHAELRFLDLVPSLQLDPAQIYRVTWFISWSPCFSWGCAGEVRAFLQENTHVRLRIFAARIYDYDPLYKEALQMLRDAGAQVSIMTYDEFEYCWDTFVYRQGCPFQPWDGLEEHSQALSGRLRAILQ**ASSGSETPGTSESATPESTGMDKKYSIGLAIGTNSVGWAVITDEYKVPSKKFKVLGNTDRHSIKKNLIGALLFDSGETAEATRLKRTARRRYTRRKNRICYLQEIFSNEMAKVDDSFFHRLEESFLVEEDKKHERHPIFGNIVDEVAYHEKYPTIYHLRKKLVDSTDKADLRLIYLALAHMIKFRGHFLIEGDLNPDNSDVDKLFIQLVQTYNQLFEENPINASGVDAKAILSARLSKSRRLENLIAQLPGEKKNGLFGNLIALSLGLTPNFKSNFDLAEDAKLQLSKDTYDDDLDNLLAQIGDQYADLFLAAKNLSDAILLSDILRVNTEITKAPLSASMIKRYDEHHQDLTLLKALVRQQLPEKYKEIFFDQSKNGYAGYIDGGASQEEFYKFIKPILEKMDGTEELLVKLNREDLLRKQRTFDNGSIPHQIHLGELHAILRRQEDFYPFLKDNREKIEKILTFRIPYYVGPLARGNSRFAWMTRKSEETITPWNFEEVVDKGASAQSFIERMTNFDKNLPNEKVLPKHSLLYEYFTVYNELTKVKYVTEGMRKPAFLSGEQKKAIVDLLFKTNRKVTVKQLKEDYFKKIECFDSVEISGVEDRFNASLGTYHDLLKIIKDKDFLDNEENEDILEDIVLTLTLFEDREMIEERLKTYAHLFDDKVMKQLKRRRYTGWGRLSRKLINGIRDKQSGKTILDFLKSDGFANRNFMQLIHDDSLTFKEDIQKAQVSGQGDSLHEHIANLAGSPAIKKGILQTVKVVDELVKVMGRHKPENIVIEMARENQTTQKGQKNSRERMKRIEEGIKELGSQILKEHPVENTQLQNEKLYLYYLQNGRDMYVDQELDINRLSDYDVDHIVPQSFLKDDSIDNKVLTRSDKNRGKSDNVPSEEVVKKMKNYWRQLLNAKLITQRKFDNLTKAERGGLSELDKAGFIKRQLVETRQITKHVAQILDSRMNTKYDENDKLIREVKVITLKSKLVSDFRKDFQFYKVREINNYHHAHDAYLNAVVGTALIKKYPKLESEFVYGDYKVYDVRKMIAKSEQEIGKATAKYFFYSNIMNFFKTEITLANGEIRKRPLIETNGETGEIVWDKGRDFATVRKVLSMPQVNIVKKTEVQTGGFSKESILPKRNSDKLIARKKDWDPKKYGGFDSPTVAYSVLVVAKVEKGKSKKLKSVKELLGITIMERSSFEKNPIDFLEAKGYKEVKKDLIIKLPKYSLFELENGRKRMLASAGELQKGNELALPSKYVNFLYLASHYEKLKGSPEDNEQKQLFVEQHKHYLDEIIEQISEFSKRVILADANLDKVLSAYNKHRDKPIREQAENIIHLFTLTNLGAPAAFKYFDTTIDRKRYTSTKEVLDATLIHQSITGLYETRIDLSQLGGDEFSGGSGGSGGS*TNLSDIIEKETGKQLVIQESILMLPEEVEEVIGNKPESDILVHTAYDESTDENVMLLTSDAPEYKPWALVIQDSNGENKIKML*SGGSGGSGGS*TNLSDIIEKETGKQLVIQESILMLPEEVEEVIGNKPESDILVHTAYDESTDENVMLLTSDAPEYKPWALVIQDSNGENKIKML*SGGSRSPKKKRKVGGGGSPKKKRKV |
| A3B-T4-nCas9-2U | GPGS**EILRYLMDPDTFTFNFNNDPLVLRRRQTYLCYEVERLDNGTWVLMDQHMGFLCNEAKNLLCGFYGRHAELRFLDLVPSLQLDPAQIYRVTWFISWSPCFSWGCAGEVRAFLQENTHVRLRIFAARIYDYDPLYKEALQMLRDAGAQVSIMTYDEFEYCWDTFVYRQGCPFQPWDGLEEHSQALSGRLRAILQ**ASGGGGSKGFSSEDKGEWKLKLDNAGNGQAVIRFLPSKNDEQAPFAILVNHGFKKNGKWYIETCSSTHGDYDSCPVCQYISKNDLYNTDNKEYSLVKRKTSYWANILVVKDPAAPENEGKVFKYRFGKKIWDKINAMIAVDVEMGETPVDVTCPWEGANFVLKVKQVSGFSNYDESKFLNQSAIPNIDDESFQKELFEQMVDLSEMTSKDKFKSFEELNTKFGQVMGTAVMGGAAATAAKKAVDSGSETPGTSESATPESTGMDKKYSIGLAIGTNSVGWAVITDEYKVPSKKFKVLGNTDRHSIKKNLIGALLFDSGETAEATRLKRTARRRYTRRKNRICYLQEIFSNEMAKVDDSFFHRLEESFLVEEDKKHERHPIFGNIVDEVAYHEKYPTIYHLRKKLVDSTDKADLRLIYLALAHMIKFRGHFLIEGDLNPDNSDVDKLFIQLVQTYNQLFEENPINASGVDAKAILSARLSKSRRLENLIAQLPGEKKNGLFGNLIALSLGLTPNFKSNFDLAEDAKLQLSKDTYDDDLDNLLAQIGDQYADLFLAAKNLSDAILLSDILRVNTEITKAPLSASMIKRYDEHHQDLTLLKALVRQQLPEKYKEIFFDQSKNGYAGYIDGGASQEEFYKFIKPILEKMDGTEELLVKLNREDLLRKQRTFDNGSIPHQIHLGELHAILRRQEDFYPFLKDNREKIEKILTFRIPYYVGPLARGNSRFAWMTRKSEETITPWNFEEVVDKGASAQSFIERMTNFDKNLPNEKVLPKHSLLYEYFTVYNELTKVKYVTEGMRKPAFLSGEQKKAIVDLLFKTNRKVTVKQLKEDYFKKIECFDSVEISGVEDRFNASLGTYHDLLKIIKDKDFLDNEENEDILEDIVLTLTLFEDREMIEERLKTYAHLFDDKVMKQLKRRRYTGWGRLSRKLINGIRDKQSGKTILDFLKSDGFANRNFMQLIHDDSLTFKEDIQKAQVSGQGDSLHEHIANLAGSPAIKKGILQTVKVVDELVKVMGRHKPENIVIEMARENQTTQKGQKNSRERMKRIEEGIKELGSQILKEHPVENTQLQNEKLYLYYLQNGRDMYVDQELDINRLSDYDVDHIVPQSFLKDDSIDNKVLTRSDKNRGKSDNVPSEEVVKKMKNYWRQLLNAKLITQRKFDNLTKAERGGLSELDKAGFIKRQLVETRQITKHVAQILDSRMNTKYDENDKLIREVKVITLKSKLVSDFRKDFQFYKVREINNYHHAHDAYLNAVVGTALIKKYPKLESEFVYGDYKVYDVRKMIAKSEQEIGKATAKYFFYSNIMNFFKTEITLANGEIRKRPLIETNGETGEIVWDKGRDFATVRKVLSMPQVNIVKKTEVQTGGFSKESILPKRNSDKLIARKKDWDPKKYGGFDSPTVAYSVLVVAKVEKGKSKKLKSVKELLGITIMERSSFEKNPIDFLEAKGYKEVKKDLIIKLPKYSLFELENGRKRMLASAGELQKGNELALPSKYVNFLYLASHYEKLKGSPEDNEQKQLFVEQHKHYLDEIIEQISEFSKRVILADANLDKVLSAYNKHRDKPIREQAENIIHLFTLTNLGAPAAFKYFDTTIDRKRYTSTKEVLDATLIHQSITGLYETRIDLSQLGGDEFSGGSGGSGGS*TNLSDIIEKETGKQLVIQESILMLPEEVEEVIGNKPESDILVHTAYDESTDENVMLLTSDAPEYKPWALVIQDSNGENKIKML*SGGSGGSGGS*TNLSDIIEKETGKQLVIQESILMLPEEVEEVIGNKPESDILVHTAYDESTDENVMLLTSDAPEYKPWALVIQDSNGENKIKML*SGGSRSPKKKRKVGGGGSPKKKRKV |
| A3B-T7-nCas9-2U | GPGS**EILRYLMDPDTFTFNFNNDPLVLRRRQTYLCYEVERLDNGTWVLMDQHMGFLCNEAKNLLCGFYGRHAELRFLDLVPSLQLDPAQIYRVTWFISWSPCFSWGCAGEVRAFLQENTHVRLRIFAARIYDYDPLYKEALQMLRDAGAQVSIMTYDEFEYCWDTFVYRQGCPFQPWDGLEEHSQALSGRLRAILQ**ASGGGMAKKIFTSALGTAEPYAYIAKPDYGNEERGFGNPRGVYKVDLTIPNKDPRCQRMVDEIVKCHEEAYAAAVEEYEANPPAVARGKKPLKPYEGDMPFFDNGDGTTTFKFKCYASFQDKKTKETKHINLVVVDSKGKKMEDVPIIGGGSKLKVKYSLVPYKWNTAVGASVKLQLESVMLVELATFGGGEDDWADEVEENGYVASGSAKASKPRVDSGSETPGTSESATPESTGMDKKYSIGLAIGTNSVGWAVITDEYKVPSKKFKVLGNTDRHSIKKNLIGALLFDSGETAEATRLKRTARRRYTRRKNRICYLQEIFSNEMAKVDDSFFHRLEESFLVEEDKKHERHPIFGNIVDEVAYHEKYPTIYHLRKKLVDSTDKADLRLIYLALAHMIKFRGHFLIEGDLNPDNSDVDKLFIQLVQTYNQLFEENPINASGVDAKAILSARLSKSRRLENLIAQLPGEKKNGLFGNLIALSLGLTPNFKSNFDLAEDAKLQLSKDTYDDDLDNLLAQIGDQYADLFLAAKNLSDAILLSDILRVNTEITKAPLSASMIKRYDEHHQDLTLLKALVRQQLPEKYKEIFFDQSKNGYAGYIDGGASQEEFYKFIKPILEKMDGTEELLVKLNREDLLRKQRTFDNGSIPHQIHLGELHAILRRQEDFYPFLKDNREKIEKILTFRIPYYVGPLARGNSRFAWMTRKSEETITPWNFEEVVDKGASAQSFIERMTNFDKNLPNEKVLPKHSLLYEYFTVYNELTKVKYVTEGMRKPAFLSGEQKKAIVDLLFKTNRKVTVKQLKEDYFKKIECFDSVEISGVEDRFNASLGTYHDLLKIIKDKDFLDNEENEDILEDIVLTLTLFEDREMIEERLKTYAHLFDDKVMKQLKRRRYTGWGRLSRKLINGIRDKQSGKTILDFLKSDGFANRNFMQLIHDDSLTFKEDIQKAQVSGQGDSLHEHIANLAGSPAIKKGILQTVKVVDELVKVMGRHKPENIVIEMARENQTTQKGQKNSRERMKRIEEGIKELGSQILKEHPVENTQLQNEKLYLYYLQNGRDMYVDQELDINRLSDYDVDHIVPQSFLKDDSIDNKVLTRSDKNRGKSDNVPSEEVVKKMKNYWRQLLNAKLITQRKFDNLTKAERGGLSELDKAGFIKRQLVETRQITKHVAQILDSRMNTKYDENDKLIREVKVITLKSKLVSDFRKDFQFYKVREINNYHHAHDAYLNAVVGTALIKKYPKLESEFVYGDYKVYDVRKMIAKSEQEIGKATAKYFFYSNIMNFFKTEITLANGEIRKRPLIETNGETGEIVWDKGRDFATVRKVLSMPQVNIVKKTEVQTGGFSKESILPKRNSDKLIARKKDWDPKKYGGFDSPTVAYSVLVVAKVEKGKSKKLKSVKELLGITIMERSSFEKNPIDFLEAKGYKEVKKDLIIKLPKYSLFELENGRKRMLASAGELQKGNELALPSKYVNFLYLASHYEKLKGSPEDNEQKQLFVEQHKHYLDEIIEQISEFSKRVILADANLDKVLSAYNKHRDKPIREQAENIIHLFTLTNLGAPAAFKYFDTTIDRKRYTSTKEVLDATLIHQSITGLYETRIDLSQLGGDEFSGGSGGSGGS*TNLSDIIEKETGKQLVIQESILMLPEEVEEVIGNKPESDILVHTAYDESTDENVMLLTSDAPEYKPWALVIQDSNGENKIKML*SGGSGGSGGS*TNLSDIIEKETGKQLVIQESILMLPEEVEEVIGNKPESDILVHTAYDESTDENVMLLTSDAPEYKPWALVIQDSNGENKIKML*SGGSRSPKKKRKVGGGGSPKKKRKV |
| **A3B**-nCas9 | GPGS**EILRYLMDPDTFTFNFNNDPLVLRRRQTYLCYEVERLDNGTWVLMDQHMGFLCNEAKNLLCGFYGRHAELRFLDLVPSLQLDPAQIYRVTWFISWSPCFSWGCAGEVRAFLQENTHVRLRIFAARIYDYDPLYKEALQMLRDAGAQVSIMTYDEFEYCWDTFVYRQGCPFQPWDGLEEHSQALSGRLRAILQ**ASSGGSSGGSSGSETPGTSESATPESSGGSSGGSPAAKRVKLDGGGGSPKKKRKVTGMDKKYSIGLAIGTNSVGWAVITDEYKVPSKKFKVLGNTDRHSIKKNLIGALLFDSGETAEATRLKRTARRRYTRRKNRICYLQEIFSNEMAKVDDSFFHRLEESFLVEEDKKHERHPIFGNIVDEVAYHEKYPTIYHLRKKLVDSTDKADLRLIYLALAHMIKFRGHFLIEGDLNPDNSDVDKLFIQLVQTYNQLFEENPINASGVDAKAILSARLSKSRRLENLIAQLPGEKKNGLFGNLIALSLGLTPNFKSNFDLAEDAKLQLSKDTYDDDLDNLLAQIGDQYADLFLAAKNLSDAILLSDILRVNTEITKAPLSASMIKRYDEHHQDLTLLKALVRQQLPEKYKEIFFDQSKNGYAGYIDGGASQEEFYKFIKPILEKMDGTEELLVKLNREDLLRKQRTFDNGSIPHQIHLGELHAILRRQEDFYPFLKDNREKIEKILTFRIPYYVGPLARGNSRFAWMTRKSEETITPWNFEEVVDKGASAQSFIERMTNFDKNLPNEKVLPKHSLLYEYFTVYNELTKVKYVTEGMRKPAFLSGEQKKAIVDLLFKTNRKVTVKQLKEDYFKKIECFDSVEISGVEDRFNASLGTYHDLLKIIKDKDFLDNEENEDILEDIVLTLTLFEDREMIEERLKTYAHLFDDKVMKQLKRRRYTGWGRLSRKLINGIRDKQSGKTILDFLKSDGFANRNFMQLIHDDSLTFKEDIQKAQVSGQGDSLHEHIANLAGSPAIKKGILQTVKVVDELVKVMGRHKPENIVIEMARENQTTQKGQKNSRERMKRIEEGIKELGSQILKEHPVENTQLQNEKLYLYYLQNGRDMYVDQELDINRLSDYDVDHIVPQSFLKDDSIDNKVLTRSDKNRGKSDNVPSEEVVKKMKNYWRQLLNAKLITQRKFDNLTKAERGGLSELDKAGFIKRQLVETRQITKHVAQILDSRMNTKYDENDKLIREVKVITLKSKLVSDFRKDFQFYKVREINNYHHAHDAYLNAVVGTALIKKYPKLESEFVYGDYKVYDVRKMIAKSEQEIGKATAKYFFYSNIMNFFKTEITLANGEIRKRPLIETNGETGEIVWDKGRDFATVRKVLSMPQVNIVKKTEVQTGGFSKESILPKRNSDKLIARKKDWDPKKYGGFDSPTVAYSVLVVAKVEKGKSKKLKSVKELLGITIMERSSFEKNPIDFLEAKGYKEVKKDLIIKLPKYSLFELENGRKRMLASAGELQKGNELALPSKYVNFLYLASHYEKLKGSPEDNEQKQLFVEQHKHYLDEIIEQISEFSKRVILADANLDKVLSAYNKHRDKPIREQAENIIHLFTLTNLGAPAAFKYFDTTIDRKRYTSTKEVLDATLIHQSITGLYETRIDLSQLGGDEFPKKKRKVGGGGSPKKKRKV |
| NT4-**A3B**-nCas9 | GPGSPAAKRVKLDKGFSSEDKGEWKLKLDNAGNGQAVIRFLPSKNDEQAPFAILVNHGFKKNGKWYIETCSSTHGDYDSCPVCQYISKNDLYNTDNKEYSLVKRKTSYWANILVVKDPAAPENEGKVFKYRFGKKIWDKINAMIAVDVEMGETPVDVTCPWEGANFVLKVKQVSGFSNYDESKFLNQSAIPNIDDESFQKELFEQMVDLSEMTSKDKFKSFEELNTKFGQVMGTAVMGGAAATAAKKAGGGGSVD**EILRYLMDPDTFTFNFNNDPLVLRRRQTYLCYEVERLDNGTWVLMDQHMGFLCNEAKNLLCGFYGRHAELRFLDLVPSLQLDPAQIYRVTWFISWSPCFSWGCAGEVRAFLQENTHVRLRIFAARIYDYDPLYKEALQMLRDAGAQVSIMTYDEFEYCWDTFVYRQGCPFQPWDGLEEHSQALSGRLRAILQ**ASSGSETPGTSESATPESTGMDKKYSIGLAIGTNSVGWAVITDEYKVPSKKFKVLGNTDRHSIKKNLIGALLFDSGETAEATRLKRTARRRYTRRKNRICYLQEIFSNEMAKVDDSFFHRLEESFLVEEDKKHERHPIFGNIVDEVAYHEKYPTIYHLRKKLVDSTDKADLRLIYLALAHMIKFRGHFLIEGDLNPDNSDVDKLFIQLVQTYNQLFEENPINASGVDAKAILSARLSKSRRLENLIAQLPGEKKNGLFGNLIALSLGLTPNFKSNFDLAEDAKLQLSKDTYDDDLDNLLAQIGDQYADLFLAAKNLSDAILLSDILRVNTEITKAPLSASMIKRYDEHHQDLTLLKALVRQQLPEKYKEIFFDQSKNGYAGYIDGGASQEEFYKFIKPILEKMDGTEELLVKLNREDLLRKQRTFDNGSIPHQIHLGELHAILRRQEDFYPFLKDNREKIEKILTFRIPYYVGPLARGNSRFAWMTRKSEETITPWNFEEVVDKGASAQSFIERMTNFDKNLPNEKVLPKHSLLYEYFTVYNELTKVKYVTEGMRKPAFLSGEQKKAIVDLLFKTNRKVTVKQLKEDYFKKIECFDSVEISGVEDRFNASLGTYHDLLKIIKDKDFLDNEENEDILEDIVLTLTLFEDREMIEERLKTYAHLFDDKVMKQLKRRRYTGWGRLSRKLINGIRDKQSGKTILDFLKSDGFANRNFMQLIHDDSLTFKEDIQKAQVSGQGDSLHEHIANLAGSPAIKKGILQTVKVVDELVKVMGRHKPENIVIEMARENQTTQKGQKNSRERMKRIEEGIKELGSQILKEHPVENTQLQNEKLYLYYLQNGRDMYVDQELDINRLSDYDVDHIVPQSFLKDDSIDNKVLTRSDKNRGKSDNVPSEEVVKKMKNYWRQLLNAKLITQRKFDNLTKAERGGLSELDKAGFIKRQLVETRQITKHVAQILDSRMNTKYDENDKLIREVKVITLKSKLVSDFRKDFQFYKVREINNYHHAHDAYLNAVVGTALIKKYPKLESEFVYGDYKVYDVRKMIAKSEQEIGKATAKYFFYSNIMNFFKTEITLANGEIRKRPLIETNGETGEIVWDKGRDFATVRKVLSMPQVNIVKKTEVQTGGFSKESILPKRNSDKLIARKKDWDPKKYGGFDSPTVAYSVLVVAKVEKGKSKKLKSVKELLGITIMERSSFEKNPIDFLEAKGYKEVKKDLIIKLPKYSLFELENGRKRMLASAGELQKGNELALPSKYVNFLYLASHYEKLKGSPEDNEQKQLFVEQHKHYLDEIIEQISEFSKRVILADANLDKVLSAYNKHRDKPIREQAENIIHLFTLTNLGAPAAFKYFDTTIDRKRYTSTKEVLDATLIHQSITGLYETRIDLSQLGGDEFPKKKRKVGGGGSPKKKRKV |
| *UGI* | *MTNLSDIIEKETGKQLVIQESILMLPEEVEEVIGNKPESDILVHTAYDESTDENVMLLTSDAPEYKPWALVIQDSNGENKIKML*SGGSGGSGGSPAAKRVKLDGGGGSPKKKRKV |

Table S2: Guide RNA and primer sequences for genomic targets.

|  | Guide sequence (5’-3’) | Primer F (5’-3’) |  |
| --- | --- | --- | --- |
| AAVS1 | GCCAGTAGCCAGCCCCGTCC | TCGTCGGCAGCGTCAGATGTGTATAAGAGACAGNNNNNNGCTGCAGCTTCCTTACACTTCC | GTCTCGTGGGCTCGGAGATGTGTATAAGAGACAGNNNNNNGAGGAATATGTCCCAGATAGCACT |
| CEL | CAGGCAGTCTTCATCCCCGT | TCGTCGGCAGCGTCAGATGTGTATAAGAGACAGNNNNNNCTGTGGGATTTCATGGAAGTTCAGC | GTCTCGTGGGCTCGGAGATGTGTATAAGAGACAGNNNNNNATCCTGGCTGGCAAGGTGG |
| EMX1-11 | GGAGCCCTTCTTCTTCTGCT | TCGTCGGCAGCGTCAGATGTGTATAAGAGACAGNNNNNNAAGCAGCACTCTGCCCTCGT | GTCTCGTGGGCTCGGAGATGTGTATAAGAGACAGNNNNNNCCCTATGTAGCCTCAGTCTTCC |
| EMX1-15 | GCTCCCATCACATCAACCGG | TCGTCGGCAGCGTCAGATGTGTATAAGAGACAGNNNNNNCCTGAGTTTCTCATCTGTGCCC | GTCTCGTGGGCTCGGAGATGTGTATAAGAGACAGNNNNNNTGACTCCAGGCCTCCCCAAA |
| HBB | CTTGCCCCACAGGGCAGTAA | TCGTCGGCAGCGTCAGATGTGTATAAGAGACAGNNNNNNGCCAATCTACTCCCAGGAGC | GTCTCGTGGGCTCGGAGATGTGTATAAGAGACAGNNNNNNAGGCAGAGAGAGTCAGTGCCTA |
| HEKSite2 | GAACACAAAGCATAGACTGC | TCGTCGGCAGCGTCAGATGTGTATAAGAGACAGNNNNNNTGAACTTCCCAAGTGAGAAGCCAG | GTCTCGTGGGCTCGGAGATGTGTATAAGAGACAGNNNNNNTGGCAGGACGTCTGCCCAAT |
| RNF2 | GTCATCTTAGTCATTACCTG | TCGTCGGCAGCGTCAGATGTGTATAAGAGACAGNNNNNNTGTTAGCCAACATACAGAAGTCAGGAATGC | GTCTCGTGGGCTCGGAGATGTGTATAAGAGACAGNNNNNNTTCAGACCATAGCACTTCCCTTCC |
| EMX1-11ot | Off-target site for guide EMX1-11 | TCGTCGGCAGCGTCAGATGTGTATAAGAGACAGNNNNNNCCTCTGCATCTGACTCCACA | GTCTCGTGGGCTCGGAGATGTGTATAAGAGACAGNNNNNNTTCTCCCTCTTTGGCCCTG |
| SaCas9 bait site | TCTGCTTCTCCAGCCCTGGC | TCGTCGGCAGCGTCAGATGTGTATAAGAGACAGNNNNNNATGTGGGCTGCCTAGAAAGG | GTCTCGTGGGCTCGGAGATGTGTATAAGAGACAGNNNNNNCCCAGCCAAACTTGTCAACC |

Table S3: Insert sequences for the editing window and dinucleotide sequence specificity assay. Dinucleotide sites are highlighted in red. The barcode is underlined. The PAM is **bolded**. V = A, C, or G.

| Plasmid name | Insert sequence (5’-3’) |
| --- | --- |
| 20-14-7T | TCACCCTCTGGTTCTTGTGTCTGGTTG**TGG** |
| 19-13-6T | GCAAATTTCGGTGTCTGTGGTCGGTTG**TGG** |
| 18-12-5T | GAGAAGTGTCGTGTTCGTGGTTCGTTG**TGG** |
| 17-11-4T | AAGATTTGTTCTGTTTCTGGTTTCTTG**TGG** |
| 16-10-3T | TGGTACTGTGTCGTTTTCGGTTGTCTG**TGG** |
| 15-9-2T | ACACTGTGTGGTCTTTGTCGTTGGTCG**TGG** |
| 14-8-1T | TAGTGGTGTGGTTCTTGTTCTTGGTTC**TGG** |
| 20-14-7V | CTCACTVCTGGTVCTTGTGVCTGGTTG**TGG** |
| 19-13-6V | TCCCGGTVCGGTGVCTGTGGVCGGTTG**TGG** |
| 18-12-5V | AGGTTTTGVCGTGTVCGTGGTVCGTTG**TGG** |
| 17-11-4V | TGACGGTGTVCTGTTVCTGGTTVCTTG**TGG** |
| 16-10-3V | TAATACTGTGVCGTTTVCGGTTGVCTG**TGG** |
| 15-9-2V | TACTTGTGTGGVCTTTGVCGTTGGVCG**TGG** |
| 14-8-1V | ACAGAATGTGGTVCTTGTVCTTGGTVC**TGG** |

Table S4: Sequences of oligos, guides, and primers for *in vitro* methylation sensitivity assay. NTS = Non-targeted (edited) strand; TS = Targeted (guide-binding) strand. Primer binding sites are in italics. Guide binding sites are bolded. The PAM is underlined. Editing window NTS cytosine residues are in red. Methylated residues are indicated as “mC.”

|  | Name | Sequence (5’-3’) |
| --- | --- | --- |
| CpG | NTS-unmethyl oligo | *CCTCCCATGGTACGACTGTT*CAT**GTTCTCGCGAGTGTAGCATA**TGGCCGCCAGATATGAATTCAGAACCGGAGGACAAAG*AGAGTGCCAGAAACCAGGAA* |
|  | TS-unmethyl oligo | *TTCCTGGTTTCTGGCACTCTC*TTTGTCCTCCGGTTCTGAATTCATATCTGGCGGCCA**TATGCTACACTCGCGAGAAC**ATG*AACAGTCGTACCATGGGAGG* |
|  | NTS-methyl oligo | *CCTCCCATGGTACGACTGTT*CAT**GTTCTmCGmCGAGTGTAGCATA**TGGCCGCCAGATATGAATTCAGAACCGGAGGACAAAG*AGAGTGCCAGAAACCAGGAA* |
|  | TS-methyl oligo | *TTCCTGGTTTCTGGCACTCTC*TTTGTCCTCCGGTTCTGAATTCATATCTGGCGGCCA**TATGCTACACTmCGmCGAGAAC**ATG*AACAGTCGTACCATGGGAGG* |
|  | Guide RNA | GTTCTCGCGAGTGTAGCATA |
|  | Primer F | TCGTCGGCAGCGTCAGATGTGTATAAGAGACAGNNNNNNCCTCCCATGGTACGACTGTT |
|  | Primer R | GTCTCGTGGGCTCGGAGATGTGTATAAGAGACAGNNNNNNTTCCTGGTTTCTGGCACTCT |
| Dcm | NTS-unmethyl oligo | *TCTTGACTCTTCGCGATGTACAT***GTCACCTGGACCAGGTAGTA**TGGCCGCCAGATATGAATTCAGAACCGGAGGACAAAG*ACAAGATGGATTGCACGCAG* |
|  | TS-unmethyl oligo | *CTGCGTGCAATCCATCTTGT*CTTTGTCCTCCGGTTCTGAATTCATATCTGGCGGCCA**TACTACCTGGTCCAGGTGAC***ATGTACATCGCGAAGAGTCAAGA* |
|  | NTS-methyl oligo | *TCTTGACTCTTCGCGATGTACAT***GTCACmCTGGACmCAGGTAGTA**TGGCCGCCAGATATGAATTCAGAACCGGAGGACAAAG*ACAAGATGGATTGCACGCAG* |
|  | TS-methyl oligo | *CTGCGTGCAATCCATCTTGT*CTTTGTCCTCCGGTTCTGAATTCATATCTGGCGGCCA**TACTACmCTGGTCmCAGGTGAC***ATGTACATCGCGAAGAGTCAAGA* |
|  | Guide RNA | GTCACCTGGACCAGGTAGTA |
|  | Primer F | TCGTCGGCAGCGTCAGATGTGTATAAGAGACAGNNNNNNTCTTGACTCTTCGCGATGTAC |
|  | Primer R | GTCTCGTGGGCTCGGAGATGTGTATAAGAGACAGNNNNNNCTGCGTGCAATCCATCTTGT |

**Supplemental Methods**

Cell culture of A549 cells

Safe Harbor Landing Pad A549 cells were obtained from Merck KGaA, Darmstadt, Germany and engineered to express catalytically inactive (“dead”) SaCas9 from the AAVS1 locus per the manufacturer’s instructions. Cells were grown at 37˚C and 5% CO_2_ in DMEM supplemented with 10% FBS and 2 mM L-glutamine.

RNP and plasmid delivery of base editors to A549 cells by nucleofection

RNP complexes were assembled as described in the main methods. Plasmids were as described in the main methods. At the time of transfection, cells were trypsinized to obtain a single‐cell suspension, washed twice with Hank’s Balanced Salt Solution and resuspended in Nucleofector Solution V (Lonza, Basel, CH) at 1.9 x 10^5^ cells per 100 μL. Nucleofection was performed by mixing 100 μL of prepared cell suspension with prepared RNP complex or plasmid by pipetting up and down six times before transferring to a cuvette for electroporation using program X‐001 on a Nucleofector 2b machine. Nucleofected cells were immediately transferred to 6‐well plates containing 2 mL pre‐warmed media per well and grown for 3 days before harvest. Cell harvest, library preparation, sequencing, and data analysis were performed as described in the main methods.

Orthologous R-loop assay utilizing non-liposomal polymer transfection reagent to deliver plasmids

Plasmids were as described in the main methods. Before beginning the experiment (Day 0), HEK293 cells were seeded at 2.5 x 10^5^ cells/well into 6-well plates and 1.25 x 10^6^ cells per T75 flask. The next day (Day 1), cells were transfected with plasmids using the *Trans*IT-CRISPR^R^ reagent (Merck KGaA, Darmstadt, Germany). Briefly, for cells in 6-well plates, 3 μg of base editor plasmid, 1 μg base editor guide plasmid, 3 μg nSaCas9 plasmid, and 1 μg SaCas9 guide plasmid were mixed with 24 μL of *Trans*IT-CRISPR reagent in 250 μL of DMEM and incubated at room temperature for 30 min. 250 μL of this mixture was added to the plated cells, and the plates were rocked gently to distribute the mix and incubated for 3 days at 37˚C before harvest.

For cells in T75 flasks, 15 μg nSaCas9 plasmid and 5 μg SaCas9 guide plasmid were mixed with 60 μL of *Trans*IT-CRISPR reagent in 1.9 mL of DMEM and incubated at room temperature for 30 min. 1.9 mL of this mixture was added to the cells in the flask, which was rocked gently to distribute the mix and incubated overnight at 37˚C.

The next day (Day 2), RNP complexes were assembled as described in the main methods. Cells in flasks that had been transfected with only the SaCas9 plasmids were nucleofected with RNPs at 2.5 x 10^5^ cells per sample and plated in 6-well plates. As appropriate, PEXBUFF was added at 0.5 μL per 100 μL of cell suspension. Cells were grown for three days at 37˚C before harvest.

Cell harvest, library preparation, sequencing, and data analysis were performed as described in the main methods.

**Supplemental Figures**

Figure S1: C-to-T editing rates by position at two additional genomic targets in HEK293 cells for CBE variants A3B-nCas9-2U, T4-A3B-nCas9-2U, and T7-A3B-nCas9-2U. Cytosine positions are numbered from the 5’ end of the target sequence. Target sequences are given below each plot, with the PAM underlined. Data for CBE samples are the average of two technical replicates. Data for the Unedited control are the average of four biological replicates. Error bars represent the standard deviation.

HBB target sequence: **C**TTG**CCCC**A**C**AGGG**C**AGTAACGG

HEKSite2 target sequence: GAA**C**A**C**AAAG**C**ATAGA**C**TG**C**GGG

Figure S2: C-to-T editing rates by target in HEK293 cells for CBE variants A3B-nCas9-2U, T4-A3B-nCas9-2U, and T7-A3B-nCas9-2U. Analysis was performed on the same read data used for Fig 1C and Fig S1. Data bars represent the standard deviation. Statistical significance in editing rate between T4-A3B-nCas9-2U and T7-A3B-nCas9-2U was calculated by t-test, with p-values indicated.


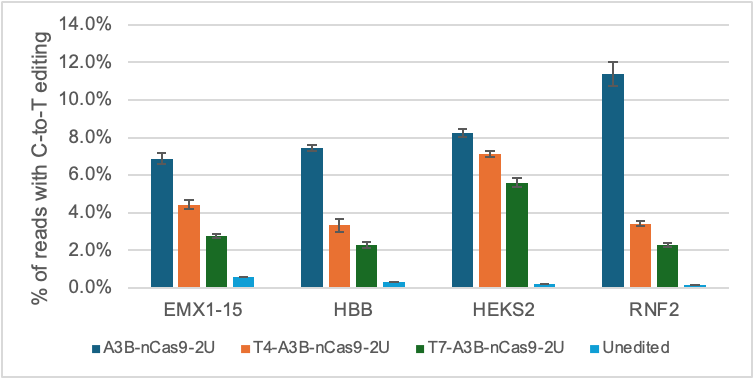


p=0.0119

p=0.0610

p=0.0181

p=0.0120

Figure S3: Presence/absence of 2x UGI in fusion protein does not affect the pattern of editing. Substitution rates by position at three genomic targets in HEKS293 cells. Portrayed are C-to-T (black), C-to-G (white), and C-to-A (gray) substitutions. Analysis uses the same data set as Figure 2A&B.

Figure S4: Co-delivery of UGI protein increases the ratio of C-to-T editing to indel formation at most genomic sites tested. Proteins were purified using gravity column chromatography and analysis uses the same read data set as used in Figure 2C-E. Data are the average of two technical replicates; error bars represent the standard deviation. Reads that contain both substitution and indel are recorded only as indels.

Figure S5: PEXBUFF transfection enhancer increases overall editing rates and can be used with UGI protein. Proteins were purified using gravity column chromatography. Base editor RNPs were delivered into HEK293 cells alone or with 7 μg UGI protein and/or PEXBUFF transfection enhancer. Data are the average of two technical replicates; error bars represent the standard deviation. Reads that contain both substitution and indel are recorded only as indels. A) Absolute rates of C-to-T editing and indel formation at genomic targets HBB and HEKSite2 for Flexible and Precision editors. R, RNP alone; R+U, RNP + UGI; R+P, RNP + PEXBUFF; R+P+U, RNP + PEXBUFF + UGI. B) Ratio of C-to-T edits to indel rates at four genomic loci. This analysis uses the same read data set as Figures 3A and S5A. C) Percent of substitutions that are C-to-T at genomic targets HBB and HEKSite2 for Flexible and Precision editors.

| A |
| --- |
|  |
| B |
|  |
| C |
|  |

Figure S6: FLPC purification of Flexible and Precision results in purer protein and higher editing rates. **Left**: Proteins were run on 4-20% gradient SDS-polyacrylamide gels at 1 μg per lane. 1, Dual Color Precision Plus protein ladder (BioRad); 2, Flexible, gravity filtration method; 3, Precision, gravity filtration method; 4, Flexible, FPLC method (replicate 1); 5, Flexible, FPLC method (replicate 2); 6, Precision, FPLC method. FPLC purification results in an increase in the expected product above the 150 kDa ladder band and removed a prominent band just above 25 kDa. **Right**: 40 pmol base editor RNP targeting genomic site EMX1-15 and 15 μg UGI protein were delivered by nucleofection into HEK293 cells and editing efficiency evaluated by sequencing. Data are average ± std dev of two technical replicates. The purification preps used were the same for both the gel and the transfection experiment (only Flexible FPLC replicate 2 was transfected).

| 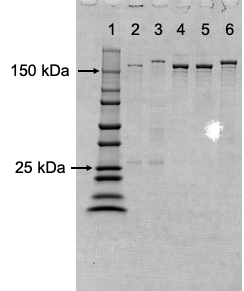 |  |
| --- | --- |

Figure S7: RNP delivery of Flexible and Precision result in fewer guide-independent off-target editing events. Absolute editing rates at the HBB on-target (top) and SaCas9 bait (bottom) sites. Editing outcomes are indicated by colored stacked bars: gray, C-to-T substitutions; white, C-to-R substitutions; black, indels. For this experiment, plasmids were delivered using a non-liposomal transfection polymer and RNPs (purified by FPLC) were delivered by nucleofection (see Supplemental Methods)

Figure S8: A549 cells are edited efficiently by Precision RNPs, but not by Precision-expressing plasmids. 40 pmol of Precision RNP targeting genomic site EMX1-11 and 15 μg of UGI were transfected by nucleofection into HEK293 cells and A549 cells stably expressing nuclease-inactivated (“dead”) SaCas9 from the AAVS1 Safe Harbor locus. In parallel, 3 μg of plasmid expressing Precision and 1 μg of plasmid expressing the EMX1-11 guide were delivered nucleofected into the same recipient cell lines. Substitution rates by position are shown as the average of two technical replicates.

Figure S9: Schematic of the *in vitro* characterization assays. For sequence context effects and editing window definition, a set of barcoded dsDNA targets were designed that, collectively, contained cytosine residues at every position within the target, in every dinucleotide context. These targets were pooled, edited *in vitro*, and the edited DNAs sequenced. For methylation, pairs of dsDNA targets were designed with and without cytosine methylation. These were edited *in vitro* and the edited DNAs sequenced.


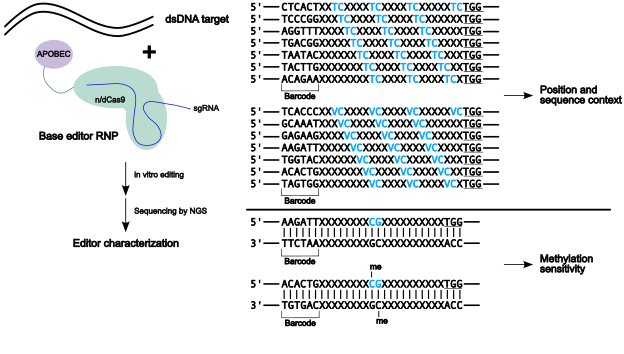


Figure S10: Methylation of cytosines on the non-target strand impedes base editing. The Dcm target sequence is given above the charts, with cytosine residues bolded and colored as in the plot. Cytosine residues that may be methylated are indicated by hashed bars. Proteins were purified by FLPC. Data for base editor conditions are the average of three technical replicates. Data for unedited controls are the average of two technical replicates, performed on separate days. Error bars represent standard deviation. Top: Absolute C-to-T editing levels of dsDNA oligo targets edited in vitro. Bottom: Normalized editing relative to the Unmethylated target. First, the rates of editing at each position were normalized to that of the control position (C3). Then, the editing rates for each test condition were normalized to those of the unmethylated target. This graph shows only cytosine residues with absolute editing levels above 1%.

Dcm methylation target sequence: GT**C**A**CC**TGGA**CC**AGGTAGTA

Figure S11: Editing at PDCD1 in cells exhibits a reduction in C-to-R substitutions at CpG sites. The target sequence is given below the plot, with cytosine residues underlined. Residues are numbered from the 5’ end of the sequence. Positions C8 and C15 are in a CpG context (bold) and exhibit an absence of C-to-R substitution. Proteins were purified by FPLC. Base editor RNPs were co-delivered to HEK293 cells with 15 μg UGI protein. Data represent the average of two technical replicates.

Target sequence: CTGGCTG**CG**GTCCT**CG**GGGA

Figure S12: *In vitro* editing of genomic sites accurately predicts editing patterns in cells. Correlation between editing rates at each cytosine position in cells (y-axis) and *in vitro* (x-axis). Editing in cells is measured as the sum of all substitutions are each cytosine position, including C-to-R. Each point represents a single cytosine residue. Plots in the left column represent data from editing by Flexible; plots in the right column represent data from editing by Precision. Data represent the average of two replicates, and error bars represent the standard deviation.

|  |  |
| --- | --- |
|  |  |
|  |  |
|  |  |
|  |  |

Figure S13: Flexible and Precision are effective in K562 cells. Proteins were purified by FPLC. Data are average and standard deviation of three replicates. A) Absolute rates of indel formation and C-to-T editing for the genomic target EMX1-15 in K562 cells. Reads that contain both substitution and indel are recorded only as indels. R, RNP; R+U, RNP + UGI; R+P, RNP + PEXBUFF; R+P+U, RNP + PEXBUFF + UGI. B) Percent of substitutions that are C-to-T.

|  | | % of substitutions that are C-to-T |
| --- | --- | --- |
| Flexible | RNP | 80.8% ± 0.2% |
|  | RNP + UGI | 97.3% ± 0.3% |
|  | RNP + PEXBUFF | 80.3% ± 0.1% |
|  | RNP + PEXBUFF + UGI | 97.5% ± 0.1% |
| Precision | RNP | 83.2% ± 0.4% |
|  | RNP + UGI | 97.9% ± 0.1% |
|  | RNP + PEXBUFF | 82.8% ± 0.2% |
|  | RNP + PEXBUFF + UGI | 98.3% ± 0.1% |

Figure S14: Flexible and Precision are effective in primary human T cells. Proteins were purified by FPLC. Data are average and standard deviation of three replicates. A) Absolute rates of indel formation and C-to-T editing for the genomic target EMX1-11 in human primary T cells. Reads that contain both substitution and indel are recorded only as indels. R, RNP; R+U, RNP + UGI; R+P, RNP + PEXBUFF; R+P+U, RNP + PEXBUFF + UGI. B) Percent of substitutions that are C-to-T. C) Top: IGV image of sequencing reads at the target site for Flexible delivered alongside UGI (editing window depicted by blue bar above the sequence). Bottom: Ratio of C-to-T editing to indel formation in primary human T cells at the genomic target EMX1-11.

| A |  |
| --- | --- |
|  |  |
| B |  |
|  | \|  \| \| % of substitutions that are C-to-T \| \| --- \| --- \| --- \| \| Flexible \| RNP \| 75.2% ± 2.7% \| \| RNP + UGI \| 90.3% ± 0.4% \| \| RNP + PEXBUFF \| 75.8% ± 1.3% \| \| RNP + PEXBUFF + UGI \| 89.3% ± 0.9% \| \| Precision \| RNP \| 72.0% ± 0.5% \| \| RNP + UGI \| 87.7% ± 1.6% \| \| RNP + PEXBUFF \| 71.8% ± 0.2% \| \| RNP + PEXBUFF + UGI \| 88.7% ± 1.9% \| |
| C |  |
|  | 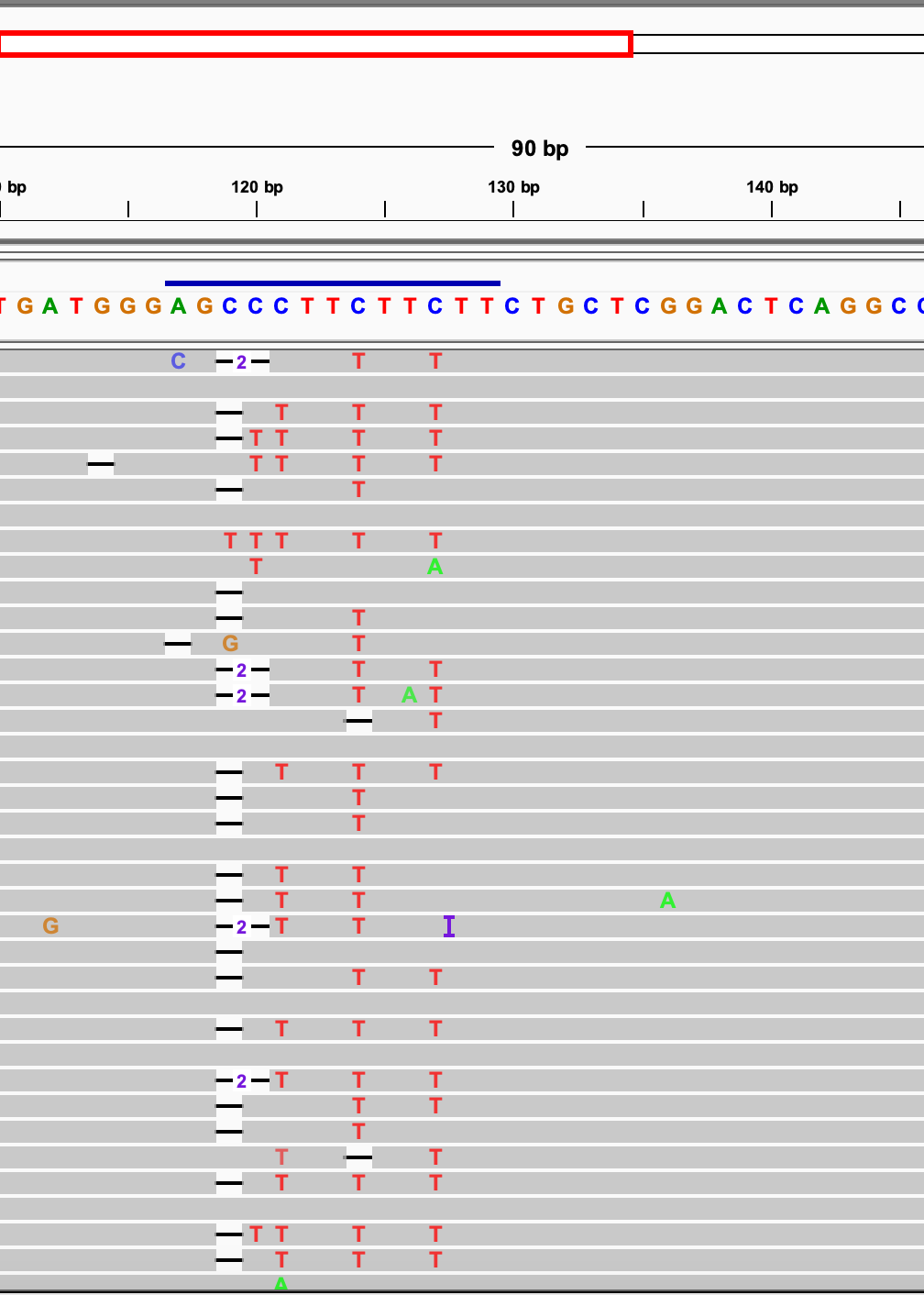 |

Figure S15: Transfection of CBE proteins does not result in loss of cell viability. Proteins were purified by FPLC and data was collected using the Cell Titer Glo 2.0 Assay. Cells used for the viability assay were drawn from the same populations as were used for NGS analysis. Data for transfections are average and standard deviation of three replicates. The Unedited control represents a single technical replicate.
